# Supplementary figures and images for: Biogeography and ecology of Ostracoda in the U.S. northern Bering, Chukchi, and Beaufort Seas
Source: PLoS One. 2021 May 13;16(5):e0251164. doi: 10.1371/journal.pone.0251164 (PMC8118254; doi:10.1371/journal.pone.0251164)

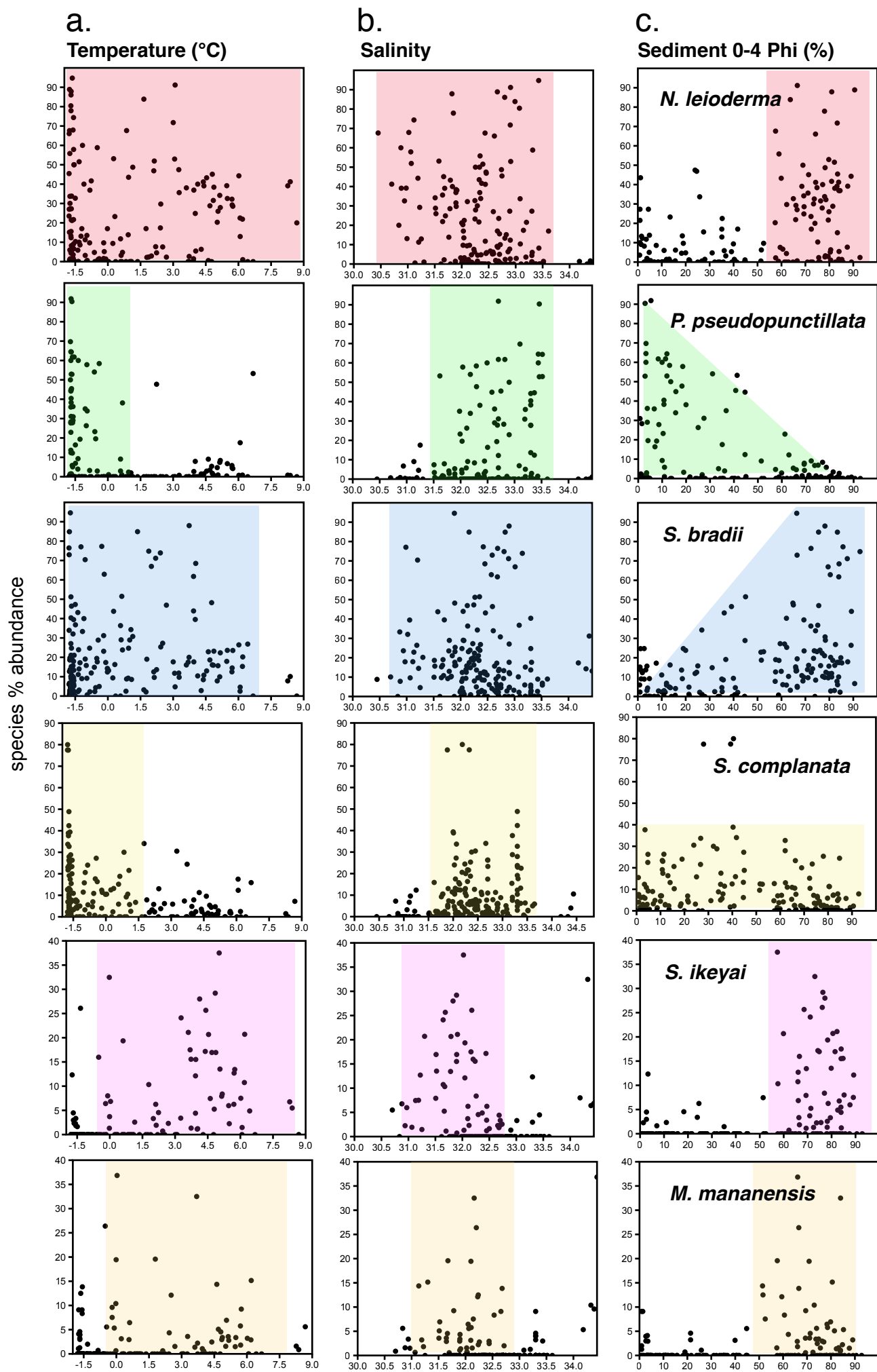

Supplement: S1 Fig — a. Faunal abundance of selected taxa in relation to near-bottom temperature during summer sediment collection in the northern Bering and Chukchi Seas (n = 211, 26,170 total specimens, 1990–2018, ≥30 specimens/sample). b. Faunal abundance in relation to salinity. c. Faunal abundance in relation to sediment type. Ostracode species abundance plotted against the percent sediment modal grain size of phi 0–4, where 0 represents gravel and rocks, 1 = coarse sand, 2 = medium sand, 3–4 = finer sand. Phi ≥5 (not shown) represents the very fine silty mud and clay sediment fraction typical of offshore or interior areas of the continental shelf. (PDF) [file pone.0251164.s001.pdf]

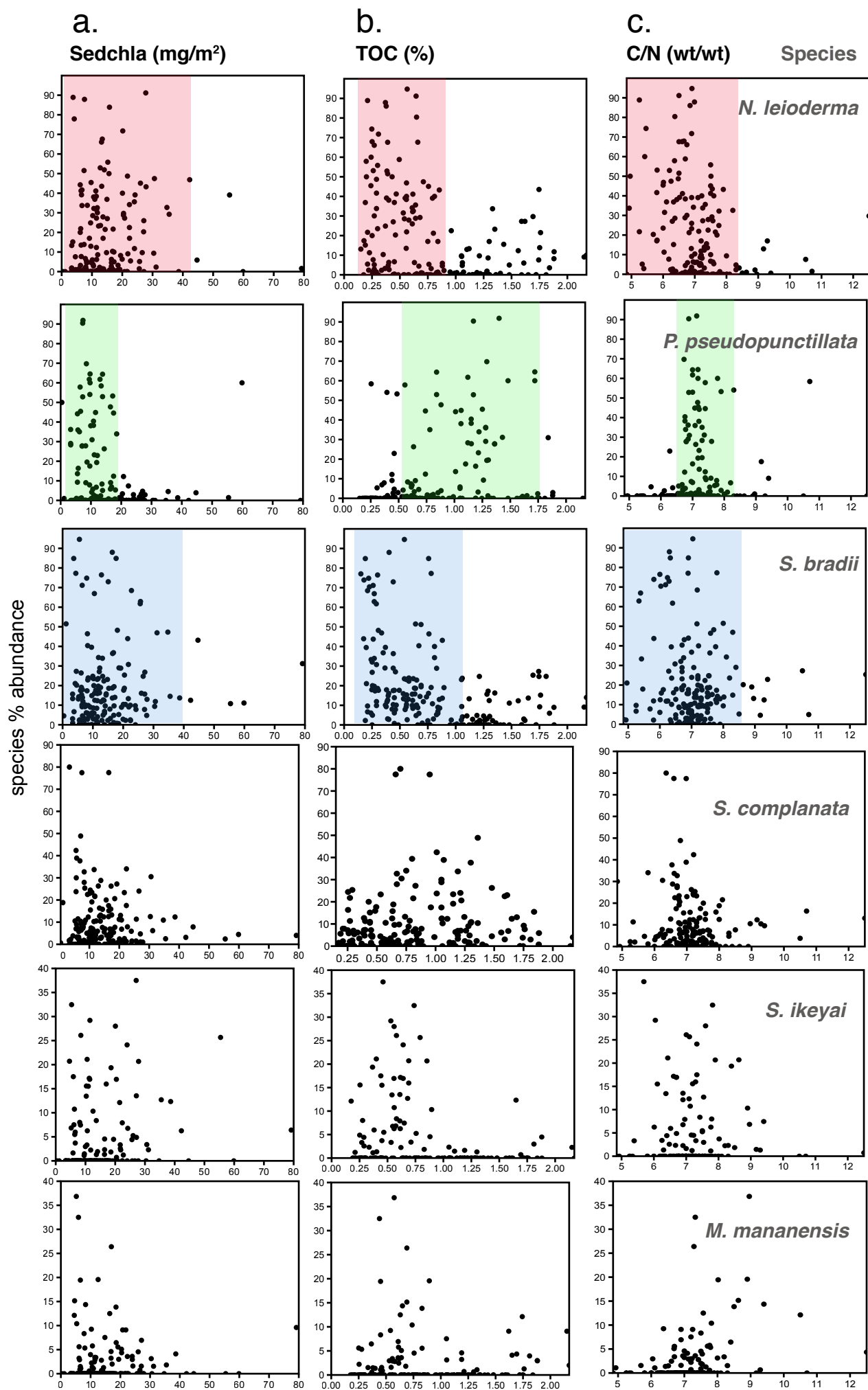

Supplement: S2 Fig — Faunal abundance plotted against three different sources of sediment carbon, which may suggest preferred food sources: a. sediment chlorophyll-a (sedchla), b. total organic carbon (TOC) and c. carbon to nitrogen (C/N) ratios. (PDF) [file pone.0251164.s002.pdf]

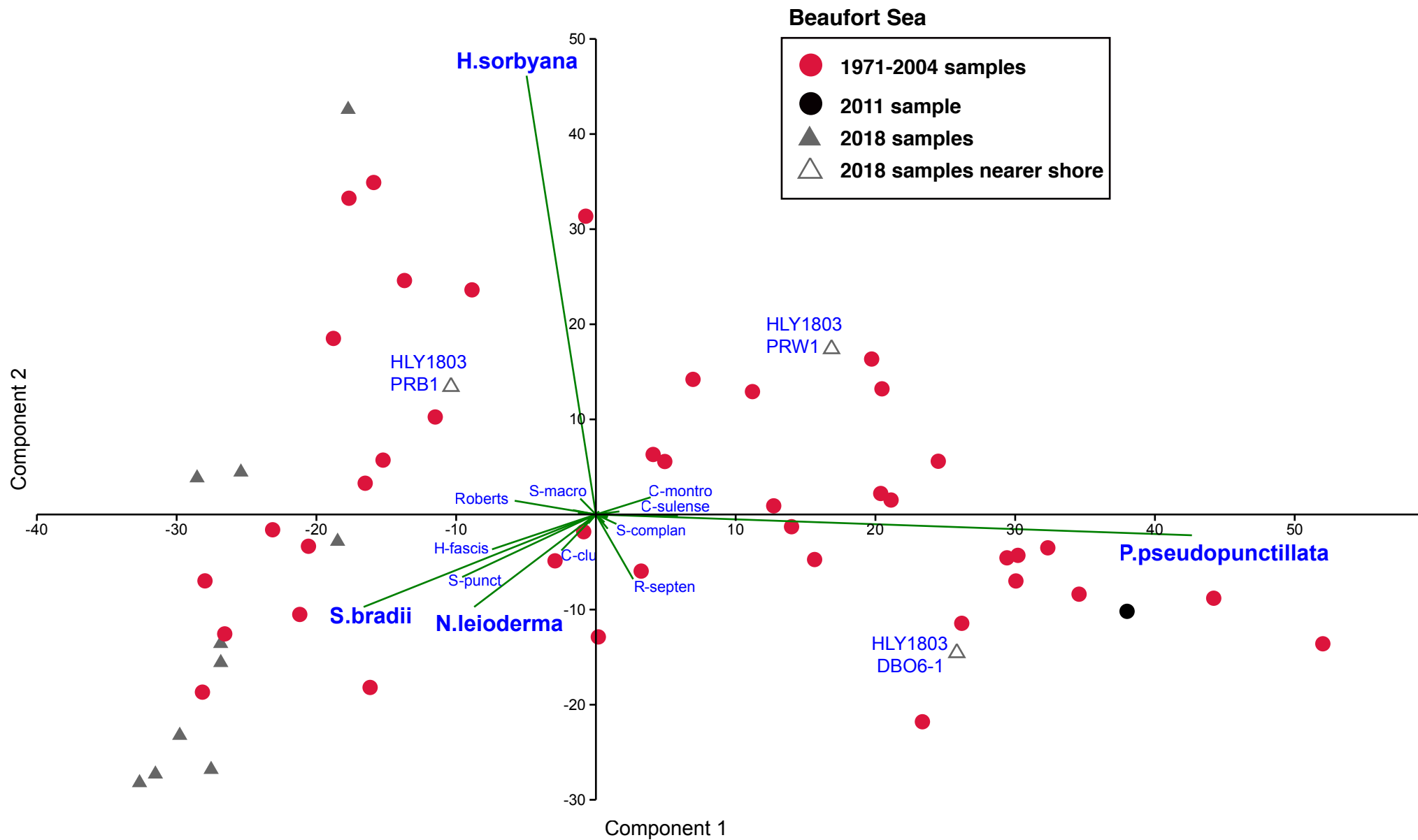

Supplement: S3 Fig — Sample sites are designated by collection years (legend symbols) and major taxa (green lines with species names labeled in blue). The dominant species in the Alaskan Beaufort Sea are H. sorbyana, P. pseudopunctillata, S. bradii and, in samples collected in 2018, N. leioderma. (PDF) [file pone.0251164.s003.pdf]
