# Supplementary material for: Biogeography and ecology of Ostracoda in the U.S. northern Bering, Chukchi, and Beaufort Seas
Source: PLoS One. 2021 May 13;16(5):e0251164. doi: 10.1371/journal.pone.0251164 (PMC8118254; doi:10.1371/journal.pone.0251164)
Supplement: S2 Table — Scanning electron microscope (SEM) photos of species are taken from Gemery et al., 2015. (PDF) [file pone.0251164.s005.pdf]

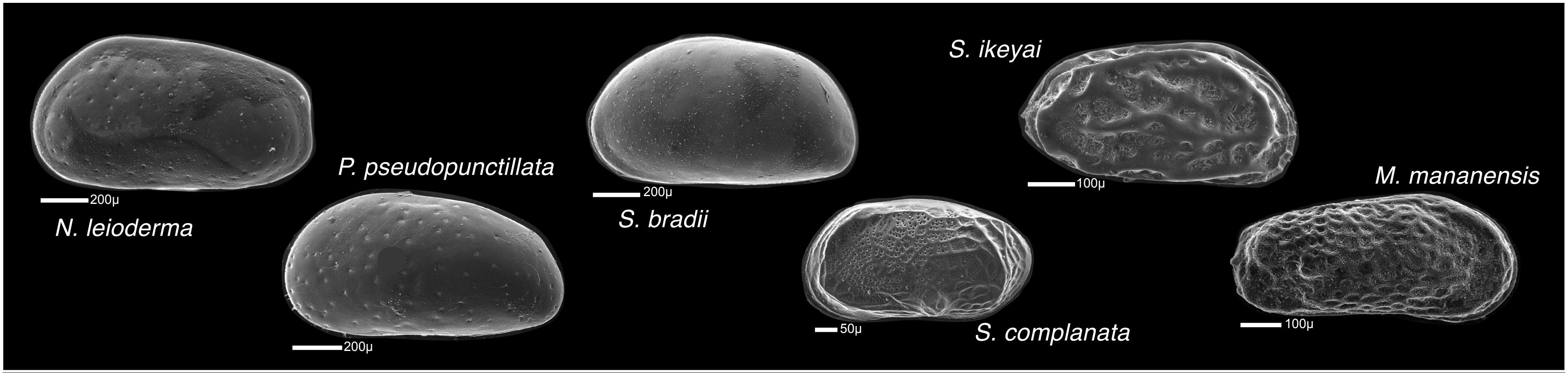

Species	ideal temperature	salinity	watermass	sediment substrate	food preference	representative climate zone	primary modern distribution*	mode of life	primary ecological reference(s)
Normanicythere leioderma	wide (-1.8 to 9°C)	nornal marine (31-33)	AW, ACW, BSW	pebbly-sandy	newly settled production	subarctic-arctic / circum-Arctic	Sea of Japan, waters off eastern Japan, Bering Sea, polyna south of St. Lawrence Island (northern Bering), Chukchi Sea, Bay areas in North Atlantic (Gulf of St. Lawrence, Nova Scotia), Ellesmere Island, Iceland, northwestern Scotland, Gulf of Maine, Spitsbergen, Russian Harbor, Novaya Zemlya (Barents Sea)	epifaunal	Hazel 1967; Ikeya and Cronin 1993; Gemery et al., 2013; this study
Paracyprideis pseudopunctillata	cold (≤1°C)	euryhaline	BSW, WW	fine silty muds	phytodetritis	arctic-subarctic / circum-Arctic	Beaufort Sea and nearshore waters off Greenland, Norwegian, Kara, Laptev, East Siberian, Norton Sound in Bering Sea, Chukchi Sea	infaunal	Stepanova, 2006
Sarsicytheridea bradii	eurythermic but more common in ≤4.5°C	nornal marine	AW, ACW, BSW	wide	wide	arctic-subarctic / circum-Arctic	North Atlantic (north of Cape Cod and Georges Bank), Ungava Bay, Frobisher Bay, Hudson Bay, Gulf of Maine, straits of the Canadian Arctic Archipelago, Newfoundland, Labrador Sea, waters off Great Britain, Ireland, Norway, Greenland, Franz Josef Land, Spitsbergen, Novaya Zemlya, White, Baltic, North, Barents, Kara, Laptev, East Siberian, Chukchi nearshore waters off Aleutian Islands, Anadyr Bay, Bering, Norton Sound, nearshore waters off Alaska	infaunal	Stepanova, 2006; Freiwald and Mostafawi, 1998; Hazel, 1970
Semicytherura complanta	cold (≤1°C)	normal marine, but high abundance in ≥33	BSW, WW	wide	unknown	arctic-subarctic / circum-Arctic	Areas where a polynya forms during wintertime; Greenland, White (southern inlet of the Barents Sea), Barents, Norwegian, Kara, Laptev, East Siberian, Chukchi, Bering, Beaufort Seas; Labrador seas, Ungava, Frobisher, and Hudson bays, straits of the Canadian Arctic Archipelago, Ireland	infaunal	Stepanova, 2006; Brouwers et al., 2000; Cronin et al. 1994
Schizocythere ikeyai	warm (0-20°C)	lower salinity areas <32	ACW	sandy	unknown	cold-temperate to subarctic	Waters off eastern Japan, Cook Inlet, Gulf of Alaska, Kodiak Shelf, Pribilof Islands, Norton Sound, Chukchi Sea	unknown	Tsukagoski and Briggs, 1998; Ozawa, 2004; Cronin et al., 2021
Munseyella mananensis	warm (0-14°C)	lower salinity areas <32	ACW	sandy	unknown	cold-temperate to subarctic	Sea of Japan, Okhost Sea, Bering Sea, Gulf of Alaska; Ungava, Frobisher, Kneeland Bays off Canada and Baffin Bay off western Greenland, Halifax Inlet off Nova Scotia	unknown	Hazel and Valentine,1969; Schornikov, 2001; 2006; Cronin et al., 2021
