# Supplementary material for: Biogeography and ecology of Ostracoda in the U.S. northern Bering, Chukchi, and Beaufort Seas
Source: PLoS One. 2021 May 13;16(5):e0251164. doi: 10.1371/journal.pone.0251164 (PMC8118254; doi:10.1371/journal.pone.0251164)

PLOS ONE

Biogeography and ecology of Ostracoda in the U.S. northern Bering, Chukchi, and Beaufort Seas

--Manuscript Draft--

**Manuscript Number:**	PONE-D-21-04622
**Article Type:**	Research Article
**Full Title:**	Biogeography and ecology of Ostracoda in the U.S. northern Bering, Chukchi, and Beaufort Seas
**Short Title:**	Biogeography and ecology of Ostracoda in the Pacific Arctic
**Corresponding Author:**	Laura Gemery<br>U.S. Department of the Interior/ U.S. Geological Survey<br>Reston, VA UNITED STATES
**Keywords:**	Ostracodes, meiobenthos, ecology, biogeography, Arctic, subarctic, cold-temperate, sublittoral, continental shelf assemblages
**Abstract:**	Ostracoda (bivalved Crustacea) comprise a significant part of the benthic meiofauna in the Pacific-Arctic region, including more than 50 species, many with identifiable ecological tolerances. These species hold potential as useful indicators of past and future ecosystem changes. In this study, we examined benthic ostracodes from nearly 300 surface sediment samples, >34,000 specimens, from three regions -- the northern Bering, Chukchi and Beaufort Seas -- to establish species' ecology and distribution. Samples were collected during various sampling programs from 1970 through 2018 on the continental shelves at 20 to ~100m water depth. Ordination analyses using species' relative frequencies identified six species, Normanicythere leioderma, Sarsicytheridea bradii, Paracyprideis pseudopunctillata, Semicytherura complanata, Schizocythere ikeyai, and Munseyella mananensis, as having diagnostic habitat ranges in bottom water temperatures, salinities, sediment substrates and/or food sources. Species relative abundances and distributions can be used to infer past bottom environmental conditions in sediment archives for paleo-reconstructions and to characterize potential changes in Pacific-Arctic ecosystems in future sampling studies. Statistical analyses further showed ostracode assemblages grouped by the summer water masses influencing the area. Offshore-to-nearshore transects of samples across different water masses showed that complex water mass characteristics, such as bottom temperature, productivity, as well as sediment texture, influenced the relative frequencies of ostracode species over small spatial scales. On the larger biogeographic scale, synoptic ordination analyses showed dominant species-- N. leioderma (Bering Sea), P. pseudopunctillata (offshore Chukchi and Beaufort Seas), and S. bradii (all regions)—remained fairly constant over recent decades. However, during 2013-2018, northern Pacific species M. mananensis and S. ikeyai increased in abundance by small but significant proportions in the Chukchi Sea region compared to earlier years. It is yet unclear if these assemblage changes signify a meiofaunal response to changing water mass properties and if this trend will continue in the future. However, our new ecological data on ostracode species and biogeography suggest these hypotheses can be tested with future benthic monitoring efforts.
**Order of Authors:**	Laura Gemery
	Thomas M. Cronin
	Lee W. Cooper
	Harry J. Dowsett
	Jackie M. Grebmeier
**Additional Information:**	
**Question**	**Response**
**Financial Disclosure**	The funders had no role in study design, data collection and analysis, decision to publish, or preparation of the manuscript.

Powered by Editorial Manager® and ProduXion Manager® from Aries Systems Corporation

Enter a financial disclosure statement that describes the sources of funding for the work included in this submission. Review the submission guidelines for detailed requirements. View published research articles from *PLOS ONE* for specific examples.<br><br>This statement is required for submission and **will appear in the published article** if the submission is accepted. Please make sure it is accurate.<br><br>**Unfunded studies**<br>Enter: *The author(s) received no specific funding for this work.*<br><br>**Funded studies**<br>Enter a statement with the following details:<br>• Initials of the authors who received each award<br>• Grant numbers awarded to each author<br>• The full name of each funder<br>• URL of each funder website<br>• Did the sponsors or funders play any role in the study design, data collection and analysis, decision to publish, or preparation of the manuscript?<br>• **NO** - Include this sentence at the end of your statement: *The funders had no role in study design, data collection and analysis, decision to publish, or preparation of the manuscript.*<br>• **YES** - Specify the role(s) played.<br><br>* typeset	
**Competing Interests**<br><br>Use the instructions below to enter a competing interest statement for this submission. On behalf of all authors, disclose any competing interests that could be perceived to bias this work—acknowledging all financial support and any other relevant financial or non-financial competing interests.<br><br>This statement is required for submission and **will appear in the published article** if the submission is accepted. Please make	The authors have declared that no competing interests exist.

sure it is accurate and that any funding sources listed in your Funding Information later in the submission form are also declared in your Financial Disclosure statement.<br><br>View published research articles from *PLOS ONE* for specific examples.<br><br>**NO authors have competing interests**<br><br>Enter: *The authors have declared that no competing interests exist.*<br><br>**Authors with competing interests**<br><br>Enter competing interest details beginning with this statement:<br><br>*I have read the journal's policy and the authors of this manuscript have the following competing interests: [insert competing interests here]*<br><br>* typeset	
**Ethics Statement**<br><br>Enter an ethics statement for this submission. This statement is required if the study involved:<br><br>• Human participants<br>• Human specimens or tissue<br>• Vertebrate animals or cephalopods<br>• Vertebrate embryos or tissues<br>• Field research<br><br>Write "N/A" if the submission does not require an ethics statement.<br><br>General guidance is provided below. Consult the submission guidelines for detailed instructions. **Make sure that all information entered here is included in the Methods section of the manuscript.**	N/A

**Format for specific study types**<br><br>**Human Subject Research (involving human participants and/or tissue)**<br>• Give the name of the institutional review board or ethics committee that approved the study<br>• Include the approval number and/or a statement indicating approval of this research<br>• Indicate the form of consent obtained (written/oral) or the reason that consent was not obtained (e.g. the data were analyzed anonymously)<br><br>**Animal Research (involving vertebrate animals, embryos or tissues)**<br>• Provide the name of the Institutional Animal Care and Use Committee (IACUC) or other relevant ethics board that reviewed the study protocol, and indicate whether they approved this research or granted a formal waiver of ethical approval<br>• Include an approval number if one was obtained<br>• If the study involved *non-human primates*, add *additional details* about animal welfare and steps taken to ameliorate suffering<br>• If anesthesia, euthanasia, or any kind of animal sacrifice is part of the study, include briefly which substances and/or methods were applied<br><br>**Field Research**<br><br>Include the following details if this study involves the collection of plant, animal, or other materials from a natural setting:<br>• Field permit number<br>• Name of the institution or relevant body that granted permission	
**Data Availability**<br><br>Authors are required to make all data underlying the findings described fully available, without restriction, and from the time of publication. PLOS allows rare exceptions to address legal and ethical concerns. See the PLOS Data Policy and FAQ for detailed information.	Yes - all data are fully available without restriction

A Data Availability Statement describing where the data can be found is required at submission. Your answers to this question constitute the Data Availability Statement and **will be published in the article**, if accepted.<br><br>**Important:** Stating ‘data available on request from the author’ is not sufficient. If your data are only available upon request, select ‘No’ for the first question and explain your exceptional situation in the text box.<br><br>Do the authors confirm that all data underlying the findings described in their manuscript are fully available without restriction?	
**Describe where the data may be found in full sentences. If you are copying our sample text, replace any instances of XXX with the appropriate details.**<br><br>• If the data are **held or will be held in a public repository**, include URLs, accession numbers or DOIs. If this information will only be available after acceptance, indicate this by ticking the box below. For example: *All XXX files are available from the XXX database (accession number(s) XXX, XXX.).*<br>• If the data are all contained **within the manuscript and/or Supporting Information files**, enter the following: *All relevant data are within the manuscript and its Supporting Information files.*<br>• If neither of these applies but you are able to provide **details of access elsewhere**, with or without limitations, please do so. For example:<br><br>*Data cannot be shared publicly because of [XXX]. Data are available from the XXX Institutional Data Access / Ethics Committee (contact via XXX) for researchers who meet the criteria for access to confidential data.*<br><br>*The data underlying the results presented in the study are available from (include the name of the third party*	AOD-2020 is available at NOAA's National Centers for Environmental Information (NCEI) World Data Service for Paleoclimatology for download (https://www.ncdc.noaa.gov/paleo/study/32312; Cronin et al., 2021). Data obtained during the DBO sampling efforts are available at the Earth Observing Laboratory (EOL) of the National Center for Atmospheric Research (NCAR), http://www.eol.ucar.edu/field_projects/dbo and at the Arctic Data Center (https://arcticdata.io/catalog/portals/DBO), supported by the National Science Foundation.

and contact information or URL).<br>• This text is appropriate if the data are owned by a third party and authors do not have permission to share the data.<br><br>* typeset	
Additional data availability information:	

Manuscript

Click here to access/download;Manuscript;Biogeo Ostracoda Final for submission2-9-2021.docx

Biogeography and Ecology of Ostracoda in the U.S. northern Bering, Chukchi, and Beaufort Seas

L. Gemery[1,2], T.M. Cronin,[1] L.W. Cooper[2], H.J. Dowsett[1], J.M. Grebmeier[2]

1. U.S. Geological Survey, Florence Bascom Geoscience Center, 12201 Sunrise Valley Drive, Reston, VA, USA
2. Chesapeake Biological Laboratory, University of Maryland Center for Environmental Sciences, Solomons, MD, USA

Corresponding author
Laura Gemery, lgemery@usgs.gov

Keywords: Ostracodes, meiobenthos, ecology, biogeography, Arctic, subarctic, cold-temperate, sublittoral, continental shelf assemblages

Abstract

Ostracoda (bivalved Crustacea) comprise a significant part of the benthic meiofauna in the Pacific-Arctic region, including more than 50 species, many with identifiable ecological tolerances. These species hold potential as useful indicators of past and future ecosystem changes. In this study, we examined benthic ostracodes from nearly 300 surface sediment samples, >34,000 specimens, from three regions -- the northern Bering, Chukchi and Beaufort Seas -- to establish species' ecology and distribution. Samples were collected during various sampling programs from 1970 through 2018 on the continental shelves at 20 to ~100m water depth. Ordination analyses using species' relative frequencies identified six species, *Normanicythere leioderma, Sarsicytheridea bradii, Paracyprideis pseudopunctillata, Semicytherura complanata, Schizocythere ikeyai,* and *Munseyella mananensis,* as having diagnostic habitat ranges in bottom water temperatures, salinities, sediment substrates and/or food sources. Species relative abundances and distributions can be used to infer past bottom environmental conditions in sediment archives for paleo-reconstructions and to characterize potential changes in Pacific-Arctic ecosystems in future sampling studies.

Statistical analyses further showed ostracode assemblages grouped by the summer water masses influencing the area. Offshore-to-nearshore transects of samples across different water masses showed that complex water mass characteristics, such as bottom temperature, productivity, as well as sediment texture, influenced the relative frequencies of ostracode species over small spatial scales. On the larger biogeographic scale, synoptic ordination analyses showed dominant species-

- *N. leioderma* (Bering Sea), *P. pseudopunctillata* (offshore Chukchi and Beaufort Seas), and *S. bradii* (all regions)—remained fairly constant over recent decades. However, during 2013-2018, northern Pacific species *M. mananensis* and *S. ikeyai* increased in abundance by small but significant proportions in the Chukchi Sea region compared to earlier years. It is yet unclear if these assemblage changes signify a meiofaunal response to changing water mass properties and if this trend will continue in the future. However, our new ecological data on ostracode species and biogeography suggest these hypotheses can be tested with future benthic monitoring efforts.

Introduction

Biological systems of the Pacific-influenced Arctic Ocean are currently undergoing rapid climate-related transformations (e.g. Huntington et al., 2020; Grebmeier et al. 2006b; Grebmeier, 2012; Wassmann et al., 2011). This is attributed to ocean warming in the North Pacific, Bering, and Chukchi Seas (Wood et al., 2015a; Woodgate, 2018), which is accelerating sea-ice loss and extending the open water season (Frey et al., 2015; Serreze et al., 2016). Changes in sea-ice cover further alter stratification, hydrography, and circulation patterns, all of which influence primary productivity (Stabeno et al., 2017; Lewis et al., 2020; Hill et al., 2018; Arrigo and van Dijken, 2015) and trophic relationships (Nelson et al., 2014; Stabeno et al., 2019). The quality, quantity, and timing of production reaching the sea floor ultimately affects benthic marine species abundance, distribution, and food web dynamics (Grebmeier, 2012; Grebmeier et al., 2006b, 2015a, 2018). In this study, we examine a component of the benthic ecosystem: meiobenthic ostracodes and the primary factors that influence their ecology and biogeography in response to changing conditions in the Bering, Chukchi, and Beaufort Seas.

Ostracodes as indicators of environmental change

Ostracodes are a bivalved group of Crustacea, ranging in size from ~0.5 to 2.0 mm, that secrete a calcareous ($CaCO_3$) shell commonly preserved in sediments. Because individual marine ostracode species have ecological limits controlled by temperature, salinity, oxygen, sea ice, food, and other habitat-related factors, they are useful ecologic indicators (e.g. Ikeya and Cronin, 1993; Frenzel and Boomer, 2005; Stepanova et al., 2003, 2007; Horne et al., 2012). Ostracodes provide ways to evaluate the effects of changing environmental conditions in different types of areas/ecotones and through geologic time. In addition to distinct species' ecology, their small size, abundant fossil record, limited stratigraphic ranges, species-level identification, and shell isotope geochemistry, ostracodes are multi-proxy tools for paleoecologic, paleoceanographic reconstructions and

biostratigraphy (e.g. Brouwers, 1992; Ikeya and Cronin, 1993; Stepanova et al., 2007; Gemery et al., 2017).

Controls on marine ecosystems and benthic ostracode species

The distribution of biological communities—from plankton and invertebrates to epibenthic fish and sea birds—has largely been found to reflect the distribution of water masses (Grebmeier et al., 2018; Sigler et al., 2017). While the temperature required for survival and reproduction primarily controls the distribution of cryophilic ostracode species on large biogeographic scales (Hazel, 1970; Hutchins, 1947), other local factors also influence species spatial dynamics, such as depth, sediment properties, and primary productivity exported to the benthos (Elofson, 1941; Ikeya and Cronin, 1993; Ozawa, 2003, 2004; Yasuhara et al., 2012). Assemblage composition in any given area is constrained by a combination of these environmental gradients. In addition to water mass properties, this study examines other parameters that affect dominant and indicator ostracode species over south to north gradients (Table 1 and Fig 1), as well as nearshore to offshore transects (Fig 2 and Fig 3), to better understand the ecological preferences of important indicator species.

Here, we extend a prior study analyzing ostracode distributions in the Bering Sea (Gemery et al., 2013), using additional samples collected through the Distributed Biological Observatory (DBO). This international hydrographic and biological sampling program, initiated in 2010, targets known areas of high biological activity along latitudinal transects in the Pacific-Arctic for repeated sampling and ecosystem monitoring (Moore and Grebmeier, 2018). We incorporated an ostracode species analysis into the DBO framework with samples collected from 1970 to 2018 and others in the vicinity of these locations (Fig 1) using a subset of data from the Arctic Ostracode Database (AOD-2020; Cronin et al., 2021; Gemery et al., 2015; Cronin et al., 2010; Cronin et al., 1991). The AOD-2020 compiles data for 96 extant ostracode species from 1574 Arctic and subarctic surface (most from the upper 0-2cm) samples, with associated data that include geographic coordinates, water depth, temperature, and salinity. This allowed multivariate analyses to be done on a subset of samples in the northern Bering, Chukchi, and Beaufort (BCB) Seas over the past several decades.

This study has three primary objectives. The first is to examine large-scale biogeographic ostracode patterns and species diversity in the BCB Seas. The second objective is to identify primary environmental factors related to ostracode faunal distributions, particularly among

dominant and diagnostic species. For this, we used ordination analyses of a series of nearshore-to-offshore transects (primarily the DBO lines) to spatially assess species abundance patterns across different water masses. Lastly, we use distribution and abundance patterns of dominant and/or ecologically significant taxa as proxies for species response to environmental changes and consider whether recent temperature, sea ice, and productivity changes are affecting ostracode distributions.

Environmental setting

The continental shelves in the northern BCB Seas include several water masses with different origins that differ in properties, including temperature, salinity, nutrients (Coachman et al., 1975). The Bering Sea is the major source of nutrients, heat, and freshwater flowing into the Chukchi Sea through the Bering Strait (Woodgate, 2018), including three distinct summer water masses: Anadyr Water, Alaska Coastal Water, and Bering Sea Water (Fig 2; Coachman et al., 1975; Weingartner et al., 2005, 2013). Anadyr Water (AW; $-1.0° < T < 1.5°$, $S \geq 32.5$) is relatively saline, cold, and nutrient-rich and flows along the Russian coast and the western side of the northern Bering Sea. Alaska Coastal Water (ACW; $3° < T < 9°$, $S \leq 32$) in summer contains warmer and fresher water derived in part from Alaskan river runoff (Weingartner et al., 2005). Bering Shelf Water (BSW; $0° < T < 3.0°$, $31.8 < S < 33$) is an intermediate water type that is lower in macro-nutrients but with higher salinity contributions from the Bering Slope Current (Coachman et al., 1975). It runs northward and bifurcates at St. Lawrence Island to enter the Bering Strait between the westerly AW and easterly ACW.

In summer, currents flowing though the Bering Strait drive oceanographic properties in the Chukchi Sea, where water is made up of ACW and Bering Sea Water (BSW), which is a combination of both Bering Shelf Water and AW (Weingartner et al, 2013; Woodgate et al., 2005a&b). Bering Sea Water has a higher salinity and nutrient levels than ACW (Walsh et al., 1989; Woodgate et al., 2005b). In the central Chukchi basin, BSW contains the highest primary production and chlorophyll standing stock (Danielson et al., 2017). This water mass follows bathymetric contours of the Chukchi Sea shelf, flowing northwest to Herald Canyon and northeast across the mid-shelf Central Channel to flank Hanna Shoal to the northern tip of Alaska (Barrow Canyon; Fig 2; Weingartner et al., 2013; Danielson et al., 2017). Year-round, when the prevailing northeast winds are weak, outflowing water from the Chukchi Sea is advected toward the eastern Canadian Basin along the Beaufort shelfbreak jet (Fig 2; Pickart et al., 2009).

These seasonal ocean currents are key in transporting nutrients and phytodetritus into the western Arctic Ocean and maintaining high productivity and biomass (Grebmeier et al., 2015a). In winter and early spring, the shelf areas are uniformly comprised of cold Winter Water (WW; T≤ -1.0°, S>31.5; Gong and Pickart, 2015; Danielson et al., 2020). In summer and fall, WW remains present in the bottom waters of the northern Chukchi Sea (Danielson et al., 2020).

Materials and methods

Sample collection and processing

The AOD-2020 compiles ostracode census data collected from circum-Arctic and subarctic sites between 1970 to 2018 from a number of programs and expeditions. Geographical coordinates, depth, temperature, salinity, and original source methodology for all samples in the database are based on original published studies. Most samples were obtained from the uppermost 0-2 cm of surface sediments. For collections during 2009-2018, including the Distributed Biological Observatory (DBO) program, surface sediments were taken from the tops of multicores or Van Veen grabs prior to opening. Most sampling occurred during the months of June, July, August, or September. Corresponding bottom water temperature and salinity were collected at the time of sampling or if shipboard expedition data were not available, as was the case for some older Beaufort Sea samples from the 1970s and 1980s, measurements were derived from World Ocean Atlas 2001 (Stephens et al., 2002; Boyer et al., 2002) and World Ocean Database 2013 (Boyer et al. 2013; as cited in Cronin et al., 2021). Salinity is given without units using the Practical Salinity Scale (Millero, 1993).

Sediments ranging in wet weight from 20 to 150g were washed with tap water though a 63μm sieve, oven-dried in paper filters at 40–50°C, and then dry sieved over a mesh-size of >125μm. In most cases, all ostracode specimens present in a sample were picked. Living and dead specimens were counted together; however, the majority of samples contained well-preserved shells and carapaces with chitinous appendages that indicated specimens were alive at or near the time of collection. The number of specimens refers to valves and articulated carapaces each counted as one specimen (Cronin et al., 1991).

Surface sediment characteristics

Because sediment composition and organic carbon content impact benthic faunal biofacies in the Pacific-Arctic (Grebmeier 2012; Grebmeier et al., 2015b), we used ancillary, corresponding surface sediment data, where available, to help characterize benthic ostracode habitat in the northern Bering and Chukchi Seas. Surface sediment total organic carbon (TOC), total organic nitrogen (TON), carbon to nitrogen ratios (C/N), sediment chlorophyll-a (sedchla, as a function of newly settled organic matter), and sediment grain size (0-4 phi = coarse pebbles to sands, ≥5 phi = fine silts) used in this study have been previously reported (see Cooper et al., 2002, 2015; Cooper and Grebmeier, 2018 and methodology therein) and are available in public data archives (PacMARS EOL data portal, http://pacmars.eol.ucar.edu/, Grebmeier and Cooper, 2014b, 2016; and Arctic Data Center, https://arcticdata.io/).

Statistical analyses

Multivariate statistical analyses were carried out using the Paleontological Statistics (PAST) software package, version 3.24 (Hammer et al., 2001). Relative frequencies (percent abundance of total assemblage) were calculated for each ostracode species. Species relative abundance data are expressed as a percentage of an individual species relative to the total number of individuals in a sample. Our studies focused on ostracode samples yielding more than 30 total specimens. Both species (R-mode) and samples (Q-mode) were grouped by several different correspondence analyses. A principal component analysis (PCA) was used to group ostracode species by their similarities/differences in dominance based on samples designated by ecoregions. A detrended correspondence analysis (DCA) described relationships between ostracode assemblage structure and geographic DBO/other ecoregions. A canonical correspondence analysis (CCA) was used to explore multivariate relationships between the most common ostracode species and their associations with major environmental variables, and sample locations were color-coded by ecoregion. The benthic environment at each station in the northern Bering and Chukchi Seas was characterized by eight variables that affect ostracode habitat and survival: bottom water temperature; bottom salinity; grain sizes 0-4 phi; grain size ≥5 phi; sediment chlorophyll-a; TOC; TON; C/N ratios. The Shannon Weaver "H" Index was used to measure ostracode diversity, a function of species richness and relative species' abundance in a community (using the formula from Legendre and Legendre, 1998). The values of "H" typically range from 0.0 to 5.0, and increases with increases in both richness (or the number of different species) and evenness (how close in abundance each species is in an environment). In general, values above 3.0 suggest a habitat structure that stable and balanced. Values less than 1.0 indicate a more challenging or impoverished habitat for survival and reproduction.

Results

Benthic ostracodes were abundant and diverse at continental shelf depths (20-~100m water depth) in Pacific Arctic surface sediments analyzed (ranging from 30 to 562 specimens/sample; Fig 1, Table 1). A total of 47 ostracode species and five genera were identified in 289 samples (34,369 total specimens) collected from 1970-2018 in the BCB Seas (Table 2). Ostracode species identified represent a mixture of Arctic, subarctic, and cold-temperate taxa. At the sampling sites, summer bottom water temperatures were highly variable, ranging from 12°C off Nome to 6-9°C near the Alaskan coast to ≤0°C in Bering and Chukchi Sea middle shelf areas (Fig 4a-e). More specifically, ecoregion comparisons of summer near-seafloor temperatures from conductivity-temperature-depth (CTD) measurements show the northern Bering Sea, southwest of St. Lawrence Island (Fig 4a, in year 2018), the Chirikov Basin (Fig 4b, red dots = DBO2 eastern-most samples off Alaska) and nearshore regions in the southeastern Chukchi Sea (Fig 4c, red dots = DBO3-1 eastern samples off Pt. Hope) recently warmed by 2-4°C above historical temperatures. Summer bottom water temperatures at sample locations in Anadyr Water in the northern Bering Sea (Fig 4b, blue diamonds) and those more centrally located in Chukchi Sea ecoregions (Fig. 4c, orange squares =DBO3-8 and Fig 4d, navy squares = DBO5-10) have not yet manifested a sustained warming trend (Grebmeier et al, 2018). The Chukchi Ecosystem Observatory (CEO) mooring, located 70 miles offshore in northeast Chukchi Sea near Hanna Shoal, (71.6 °N and 161.5 °W) rarely recorded near-seafloor temperatures above 0°C prior to 2016. That year and each following year it recorded several months with temperatures exceeding 0°C, which lasted up to four months in 2018 (Huntington et al., 2020). Bottom water temperatures representing a similar but wider geographic area in 2015 and 2017 corroborate this finding (Fig 4e).

Salinity was relatively uniform across the sampling locations in that marine conditions prevailed and averaged 32 ±2. In the Alaskan Beaufort Sea (20-90m water depth), summer temperatures across all years of sampling averaged 0°C ±1°C, and salinity averaged 31±1. Coarser, sandy sediments that occur in the hydrodynamically active nearshore areas did not cause lower ostracode abundances, as might have been expected (Kornicker, 1959; Kornicker and Wise, 1960).

Diversity “H” in the Chukchi Sea ranged from 1.8 to a little more than 2 during most sample years. Samples averaged 12 (±4) different species. The Beaufort Sea samples had comparable diversity values of 1.8, which was consistent in years 1970-2018 where sampling occurred, and 11 (±3) distinct species per sample. Ostracode diversity in the northern Bering Sea was the lowest compared to

these regions, and averaged 1.1-1.2 during the period 2009-2018 with an average of 6 (±2) species per sample.

Large-scale biogeography of ostracodes in the Bering, Chukchi, Beaufort Seas

Three dominant species in BCB Seas region accounted for 20% to 50% of the total population in the study area: *Normanicythere leioderma, Sarsicytheridea bradii*, and *Paracyprideis pseudopunctillata* (Fig 5). Each of these species clearly groups with sampling regions designated by DBO ecoregions (noted by symbol on legend) in separate quadrants of the PCA (Fig 5). In some samples along the DBO3 and central Chukchi Sea (Ledyard Bay) transects (indicated by orange squares on the PCA), *N. leioderma* and *S. bradii* were co-dominant. Otherwise, all three taxa showed distinct spatial distribution patterns related to the path of the primary summer bottom water masses (and its respective properties) in the surveyed regions. Other ostracode species (labeled in blue around the center axes on the PCA) are secondary components in assemblages.

A DCA (Fig 6a) grouped ostracode samples in four distinct geographical groups (Table 3). These ostracode biofacies are associated with specific bottom water masses, sediment substrates and/or food sources: Group 1 is a subarctic assemblage dominated by *N. leioderma* in the vicinity of the St. Lawrence Island polynya and the western Chirikov Basin at DBO2 that is influenced in summer by Anadyr Water. Group 2 is an Arctic-subarctic assemblage dominated by *S. bradii* and *N. leioderma* found in the eastern Chirikov Basin of DBO2 and nearshore areas in the southeast Chukchi Sea. Group 2 and Group 3 is bridged by *S. bradii*. In sediments further offshore in the central and middle shelf areas, *P. pseudopunctillata* was the most abundant species in Group 3, influenced by the BSW of the northeast Chukchi Sea. This offshore sample group includes sediments of Hanna Shoal, which is characterized by stable, cold bottom water temperatures and higher salinity from brine rejection, with WW present in bottom waters year-round (Danielson et al., 2020). Group 4 are the Beaufort Sea samples where influences by river influx and sea ice are strong, and ostracodes are dominated by *P. pseudopunctillata* and *H. sorbyana*.

Environmental factors influencing 14 of the most abundant ostracode species in the northern Bering and Chukchi Seas were examined by CCA (Fig 6b) and indicate that variables other than temperature and salinity water mass characteristics influence species distribution patterns. The environmental variables best correlated to ostracode assemblage structure at nearshore stations in the Chukchi Sea (orange squares in Figure 6b, i.e. DBO3-1 to 3-4, Ledyard Bay, and blue triangles at DBO5-1 to 5-3)

are coarse, sandy sediments (0-4phi) and increased temperatures of ACW. The species most associated with these characteristics include *Schizocythere ikeyai, Munseyella mananensis*, *Semicytherura mainensis,* and *Elofsonella concinna*. In addition, *N. leioderma* and *S. bradii* are associated with these regions where sediments have sandy-pebbly textures and also at some locations with high sediment chlorophyll-a in the northern Bering Sea, both south of St. Lawrence Island and in the Chirikov Basin. In samples located in the offshore northern Chukchi Sea and Hanna Shoal region (red inverted triangles and red circles respectively in Figure 6b), sustained cold temperatures (<0°C), high salinity from brine rejection during ice formation, and finer grained sediments best align to ostracode assemblage structure and the dominant species, *P. pseudopunctillata, Jonesia acuminata, Acanthocythereis dunelmensis, Robertsonites tuberculatus, Kotoracythere arctoborealis*, and *Heterocyprideis fascis*. Other biologically important variables such as TOC and TON are also correlated to this assemblage structure.

The CCA yielded four axes that explained 84.4% of the variance in the relationships between ostracode assemblage structure and environmental properties. The first two axes alone explained about 52% of the variance in the data. The CCA affirms that in the northern Bering and Chukchi Sea region, continental shelf ostracode taxa are more correlated to variables associated with distance from shore and water mass than to latitude (60-71°N). These factors include sediment substrate and food type or availability which link certain species to specific environments.

Based on ordination and multivariate results of ostracode species in the study region (Figs. 5 and 6b), we consider six species, *N. leioderma, S. bradii, P. pseudopunctillata, S. complanata, S. ikeyai*, and *M. mananensis*, to be most diagnostic of specific ranges in bottom water temperatures, salinities, sediment substrates and/or food sources (Figs. 5 and 6a&b; Supplementary Table 1; Supplementary Figs 1 and 2).

Ecoregions and transects: ecological-scale ostracode assemblages

We also incorporated DBO transect lines (Figs 2 and 3) in each ecoregion (Table 1) into our analysis of ecological preferences of dominant and ecologically significant species. Ecoregion-scale plots of species abundances across seven transects over multiple sampling years help illustrate the regional variation of ostracode biofacies and their spatial composition from nearshore to offshore (Figs 7-13). Ecoregion DBO-2 in the Chirikov Basin of the northern Bering Sea (Fig 7) shows a sharp faunal boundary between *N. leioderma* overlain by Anadyr Water on the western side of the Basin and *S. bradii* in Bering Shelf Water on the eastern side. The southeast DBO-3 ecoregion is dominated by *N.*

leioderma, S. bradii and *S. ikeyai* in ACW. In samples directly north of the Bering Strait in BSW, *S. complanata* increases in proportion among these species (Fig 8). More complex onshore-to-offshore assemblage changes are reflected in the Ledyard Bay and Icy Cape transects (Figs. 9 and 10) from 2018, both in the Chukchi Sea. Ledyard Bay and Ice Cape also show clear onshore-to-offshore changes in dominant species. With the exception of a few samples, the Hanna Shoal in the northeast Chukchi Sea ecoregion (Fig 11) is dominated by *P. pseudopunctillata* and Group 3 species (Table 3). The Barrow Canyon transect (Fig 12) shows perhaps the most complex faunal patterns reflecting the temporally and spatially complex and variable ocean water masses and depth changes in the Canyon. The Beaufort Sea west-to-east transect (Fig 13, sample locations on Fig 4 map) shows fairly consistent dominance of *H. sorbyana*, *P. pseudopunctillata*, *S. bradii* and recent contributions from *N. leioderma,* which is in sharp contrast to its abundant presence in samples of the western Chirikov Basin and southernmost Chukchi Sea. Biogeographic patterns of these species are further discussed below in the "Ecological transects" section.

Time-series analysis

Two time periods (1970-2012 and 2013-2018) were assessed by PCA to evaluate possible temporal changes in ostracode assemblages (Fig 14). These groupings were chosen based on the documentation of accelerated environmental changes (Huntington et al., 2020; Grebmeier et al., 2018) during the latter years, including sea ice duration (Frey et al., 2015), ocean temperature and oceanography (Danielson et al., 2020; Woodgate et al., 2018) and primary productivity (Frey et al., 2020). We did not find that the three most dominant species, *N. leioderma* (Bering Sea), *P. pseudopunctillata* (offshore Chukchi and Beaufort Seas) and *S. bradii* (all regions), changed in relative abundance. For the more recent 2013-2018 period, northern Pacific species *Munseyella mananensis* and *Schizocythere ikeyai* exhibited small but ecologically significant increases in abundance (~2%) and frequency in the Chukchi Sea region. Based on this evidence, we examined times series of relative frequencies of these five species, *N. leioderma, S. bradii, P. pseudopunctillata, S. ikeyai* and *M. mananensis* (Fig 15). The goal was to augment the synoptic time slice analyses to determine if the sampling was complete enough to detect decadal trends from interannual variability. The time series was limited to the last 18 years in Chukchi Sea (2000-2018) because too few repeat observations at similar locations to assess benthic change regionally.

Discussion

Distribution and ecology of dominant and indicator taxa

Normanicythere leioderma and *S. bradii* are ubiquitous in this region, with very broad environmental tolerances. These species are adapted to the large seasonal variations of the inner and middle continental shelf. *S. bradii* is a wide-ranging, cold temperate to frigid species with temperature tolerances of -1.7 to 18°C (Hazel, 1970), but it demonstrates a preference for mid-shelf depths in temperatures colder than 4.5°C and typical Arctic marine salinities on continental shelves (31-33 is typical; Gemery et al., 2015; S1 a&b Fig). Likewise, *N. leioderma* has wide temperature tolerances (-1.7 to 19°C; Hazel, 1970) in typical Arctic shelf salinity (S1 a&b Fig). As a dominant species in the northern Bering Sea (Gemery et al., 2013), its distribution is circum-Arctic, including the Chukchi, Beaufort, and Eastern Siberian Seas (Gemery et al., 2015), but is very rare in the Kara Sea and absent in the Laptev Sea (Stepanova et al., 2007). *Paracyprideis pseudopunctillata* is a cryophilic, littoral-sublittoral species (1-50m) with a southerly range limit of ~63°N latitude, tolerant of salinities as low as 5-10 (Hazel, 1970; Neale and Howe, 1975; Stepanova et al., 2003, Stepanova, 2006). *S. complanata* is adapted to the coldest conditions on continental shelves, including where polynyas form during winter (Stepanova, 2006). An analysis of this species in AOD-2020 reveals that its distribution is tied to near-freezing water temperatures ≤0°C in sublittoral depths, even though it can withstand higher seasonal temperatures.

These four species, which dominate the study area, are ideally adapted to bottom water temperatures that normally range from −1.8°C to ~4°C across the area (S1 Table). This temperature range coincides with the temperature window established for benthic macrofaunal animals prevalent at hotspot areas of the Pacific Arctic (Grebmeier et al., 2015a, 2015b).

S. ikeyai and *M. mananensis* are members of genera endemic to the Pacific that have modern distributions off eastern Japan and the Okhotsk Sea and are adapted to a wider range of summer bottom water temperatures (0-20°C; Ozawa, 2004; Schornikov, 2001) than the species discussed above. *M. mananensis* is also common to the cold temperate North Atlantic with bottom water temperatures from 2° to 14°C and water depths from 24 to 261m (Hazel and Valentine, 1969). Siddiqui and Grigg (1975) included this species in a list of sublittoral fauna from Halifax Harbour, Nova Scotia. Using the AOD-2020 to assess the subarctic-Arctic distribution of these species in samples with 10 or more specimens, *M. mananensis* is most commonly found in estuarine inlets, with highest abundances (2-30%) in Hudson Bay, North Star Bay (Thule, Greenland), Norton Sound (Alaska) and Chaunskaya Gulf (Eastern Siberian Sea) in bottom water temperatures ranging in summer from 0 to 7°C and salinities from 26 to 33. *S. ikeyai* is rare (one sample reported) in the

Beaufort Sea before 2018. It is recorded on the Chukchi Sea shelf in 8 samples (abundance of 2-38%) before 2014 in summer bottom water temperatures from 0 °C to 6°C and salinities from 31 to 33. Single specimens occurred in three samples in the Alaskan Beaufort Sea from 1971 to 1982. In the northern Bering Sea, *S. ikeyai* is rare (1.2% of the cumulative assemblage in samples collected during 2010-2018), and *M. mananensis* is extremely rare (<1% of assemblage). Increased frequency of these species in the Arctic could reflect warming bottom water temperatures since these species reach peak abundances in lower-latitude waters that generally remain above 0°C throughout most of the year. Overall, the abundance and distribution of these six species can be used to infer benthic environmental conditions and potential change in the Pacific Arctic in future studies and in sediment archives for paleo-reconstructions.

While temperature of the bottom water mass is a dominant control on ostracode distributions (Fig 6a), the CCA (Fig 6b) showed that some species are correlated to a particular sediment texture, and type/availability of carbon food sources. For a few species, *N. leioderma* and *P. pseudopunctillata*, these factors appear to drive their abundance, as supported by the transect analyses (see "Ecoregion transects" Discussion). For example, in the northern Bering and Chukchi Seas, *N. leioderma* more commonly inhabits coarse and pebbly sediments (0-4 phi) of faster moving water, as does *S. bradii, S. ikeyai,* and *M. mananensis* (Fig 6b; S1c Fig). *Paracyprideis pseudopunctillata* is found in greater proportions in silty, fine-grained seafloor habitats (≥5 phi) as indicated by its low abundance in sediments of phi 0-4 (Fig 6b; S1c Fig). *S. complanata* does not demonstrate a sediment preference (S1c Fig).

Availability of organic carbon is a top-level factor that drives species survival and reproduction (Joy and Clark, 1977). *N. leioderma* is associated with areas of newly settled organic material (i.e. higher quality carbon), as indicated by its relative abundance in areas of higher sediment chlorophyll-a (>10mg/m^2) and lower C/N ratios (<7; Fig 6b; S2a&b Fig). In contrast, *P. pseudopunctillata* resides in areas with lower sediment chlorophyll-a and higher TOC (>0.5%; indicating a greater amount of detritus in the surface sediments; Fig 6b; S2b Fig) and C/N values (>7; indicating greater terrigenous inputs; Fig 6b; S2c Fig). This may indicate *P. pseudopunctillata* uses detritus or more refractory carbon sources for food. The widespread abundance of *S. bradii* in areas of high- and low-quality carbon further supports its eurytopic nature, and it demonstrates no clear carbon (food) preference (S2a&b&c Fig). Because *S. complanata*, *M. mananensis* and *S. ikeyai* are secondary components in assemblages, their proportions are lower and therefore their environmental preferences are more difficult to evaluate. *M. mananensis* and *S. ikeyai* are present in sediments with higher C/N ratios, which corresponds to their higher proportions at sampling stations nearer to shore in ACW, e.g. southern Chukchi Sea stations DBO3-1 to DBO3-4 and northern Chukchi Sea stations DBO5-1 to

DBO5-3 (S2c Fig). In these nearshore sediments, coarse grains and gravel may dominant, but there is fine sediment in between the large grain size. This indicates variable settling rates over various seasons, providing complexity to nearshore statistical findings. Additional sampling of these species and corresponding sediments are required to better evaluate the role that sediment and organic carbon sources may have on their abundance.

Ecoregion transects: spatial relationships among ostracode biofacies

Stack plots showing the primary species in sample assemblages in an ecoregion and/or along a sampling transect (Figs. 7-13) provide insight into factors that control species occurrence and frequency. Generally in the study area, bottom water temperature declines with increasing latitude and distance from shore. Sediment composition also becomes finer-grained (greater silt fraction) with increasing distance from shore (Feder et al., 1994; Grebmeier et al., 2015a). Other properties, especially in the central and northeast Chukchi Sea, involve more complex spatial patterns (Grebmeier et al., 2015b; Blanchard et al., 2013). Varying seafloor bathymetry, sediment substrate, flux of organic carbon settling to the seafloor, and current velocities combine with water mass characteristics to create a complex habitat of varying ecological gradients. These transects show the variability of species occupying a sampling area of $0.1m^2$ at a given location on the seafloor during a summer’s day collection. At DBO2-Chirikov Basin (Fig 7a), Ledyard Bay (Fig 9), Icy Cape (Fig 10) and DBO5-Barrow Canyon (Fig 12) transects, ostracode biofacies do not show a continuous composition but instead a faunal transition from nearshore to offshore stations. The ecoregion plots show distinct abundance changes in indicator species, which were sometimes acutely distinct, based on spatial environmental changes in water mass, sediment properties and food type. These descriptions of ostracode faunal assemblages can serve as a baseline to examine future meiofaunal changes in the BCB Seas.

Chirikov Basin - DBO2

The DBO2 transect in the Chirikov Basin (Fig 7a) is an example of ostracode indicator species clearly divided by water masses of differing productivity. Stations DBO2-1 and 2-4 are the western-most locations, and are in the path of AW (Fig 2). Major differences between the water masses include higher average values of sediment chlorophyll-a and salinity (Fig 7b) to the west because the more eastern samples are underlain by BSW or a combination of ACW above and BSW below. Integrated primary production estimates in the Chirikov Basin range from ~80 g C m^{-2} yr^{-1} on the interior shelf to up to 480 g C m^{-2} yr^{-1} in AW (Hill et al., 2018). *N. leioderma* (red) is more prevalent in AW. The

eastern side of the Basin (stations DBO2-2 and 2-5) is dominated by *S. bradii* (blue) and *E. concinna* (brown), with increasing numbers of *S. ikeyai* (light blue) in 2018 samples that may reflect slightly higher average bottom water temperatures (nearly 2°C, Fig 4b). The abrupt change in faunas from east to west related to water mass channels is very consistent on interannual timescales at these sampling locations, with the exception of DBO2-4. Earlier samples from years 2003 and 2001 do not have *N. leioderma* as the dominant fauna at DBO2-4, and samples from years 2018 and 2016 do. We speculate that *N. leioderma* may have expanded its population perhaps due to increasing carbon export to the benthos and/or changes in current flow that affected sediment grain size. Since satellite monitoring began in 1979, the length of the ice-free season in the Chirikov Basin has increased by ~25 days, stimulating greater primary productivity due to a longer growing season (Brown and Arrigo, 2012; Brown et al., 2011). With *N. leioderma* as the established dominant fauna in the northern Bering Sea around the St. Lawrence Island polynya (Gemery et al., 2013), additional light and productivity may be factors that contribute to its expansion. Another major feature of this area is strong currents that create coarse seafloor sediments consisting of primarily (>75%) 0-4 phi-sized sand, which *N. leioderma* favors. Macrofaunal biomass associated with finer-grained sediments has significantly declined at sampling station DBO2.4 from 1999-2015 (Grebmeier et al., 2018). At this site, *N. leioderma* comprised >50% of the assemblage composition in 2018 and 2016 compared to <10% in 2001 and 2004 (Fig 7a).

Southeast Chukchi Sea - DBO3

At the nearest-to-shore stations, DBO3-1 to 3-4, the ostracode biofacies during 2014 to 2018 have been extremely consistent, not only in species present but species proportions as well (Fig 8). Species at DBO3-1 to 3-4 stations are in the path of ACW (temperatures 6°–10°C) that reach to the seafloor (Wood et al., 2015b). Higher sediment grain sizes at these near shore stations are consistent with the presence of the ACC. These nearshore stations experience great variability in seasonal bottom water temperatures (~10°C) and are dominated by *N. leioderma* (35%) with secondary taxa *S. bradii* (14%), *S. ikeyai* (13%), *E. concinna* (6%), *M. mananensis* (3%) and *Finmarchinella* spp. (5%). *Paracyprideis pseudopunctillata* is also present in many sample years in low abundance (2%). *H. sorbyana* (3%), an extremely euryhaline species (Cronin, 1977; Neale and Howe, 1975), appears only at nearest-to-shore DBO3-1 sites, and reflects the fresher water (average 31 salinity) of ACW. DBO3-1 to 3-4 are hotspots of ostracode abundance (samples averaged 235 specimens/sample) compared to sites further south or north. Despite high current velocities at these near shore stations, as indicated by coarser sediment grain sizes (75% phi 0-4 [±9]), sediment chlorophyll-a averaged 18

mg/m^2 (±11) at DBO3-1 to DBO3-5 sites from years 2014 to 2018, which supported consistently high abundances of *N. leioderma* and other ostracode species.

Samples just north of the Bering Strait (denoted as “N of St” on Fig 12) and DBO3-5 are offshore in a different summer water mass than DBO3 sites closer to shore. This water mass change is indicated by a different ostracode biofacies with fewer species overall and high proportions of *N. leioderma* and *Semicytherura* spp., particularly *S. complanata* and *S. mainensis*.

Ostracodes are rare or absent in sites further offshore (DBO3-6 to DBO3-8) in the path of BSW as sediment composition shifts to finer-grained sediments (slower current speeds, ~50-93% ≥5 phi) and higher TOC (>1%) values. This pattern is consistent over multiple years of sampling. Benthic macrofaunal biomass is very high at these offshore sites, with abundant production settling to the sediments (Grebmeier et al., 2015b).

Central Chukchi Sea - Ledyard Bay 2018

While the ostracode biofacies at most sampling sites along the 2018 Ledyard Bay transect are similar to those at DBO3 sites (except *S. ikeyai* is lacking at Ledyard Bay [2 individuals]), the Central Channel bearing BSW north to the shelf break (Fig 2) cuts across this sampling transect and creates sediment grain size, production and temperature gradients between the sample locations that drastically change ostracode assemblage composition. The station LB-5, in the path of ACW proximal to shore, has coarse sediments (75% 0-4 phi), a summer bottom water temperature at the time of sampling of 8.7°C, and 31 salinity. Subarctic species *N. leioderma* (20%), *M. mananensis* (6%) and *Semicytherura* species (32%) occupied this site (Fig 9). At the next site, LB-7, sediments change to finer silt (59% ≥5 phi) and *P. pseudopunctillata* becomes dominant with *Palmenella limicola* and *S. complanta. N. leioderma* is absent. Further offshore there is a grain size shift back to coarser sediments east of Herald Shoal (62% in the 0-4 phi category at LB-13, and of that, 25% in the 0-2 phi category) where *N. leioderma, S. bradii, M. mananensis* and *Kotoracythere arctoborealis* become predominant. In addition, the highest primary productivity rates in the Chukchi Sea are consistently observed in the central part of the shelf along the dividing line between the warm Alaskan Coastal Current to the east and in the colder nutrient-rich Bering shelf waters to the west (Woodgate et al., 2015). *N. leioderma’s* increasing abundance at LB-11 and LB-13 may reflect a fresh, more ample food source. TOC increases toward the offshore stations and sediment chlorophyll-a, although seasonally and yearly highly variable is highest at LB-13 (20 mg/m^2) as is *N. leioderma’s* (30%) abundance. Along the transect, temperature declined with distance from the coast from 8.7°C at LB-5

to 2.4°C at LB-13, where *N. leioderma, S. bradii, S. complanata, M. mananensis* and *K. arctoborealis* persist. This transect may be an example of how subtle changes in bathymetry and current flow can result in changes in food availability and sediment texture in very localized areas that affect the spatial distribution of benthic communities (Blanchard et al., 2013) and, likewise, ostracode biofacies.

Northeast Chukchi Sea - Icy Cape 2018

The 2018 Icy Cape transect shows the influence of bottom water mass and sediment texture on assemblage composition. Bottom water temperature closest to shore at ICY2 was 1.4°C, but more offshore stations were colder with temperatures of 0° to -1°C (Fig 10). In the northeast Chukchi Sea, sea-ice coverage usually extends into the summer months (July- August), with areas of localized persistence possible throughout the summer. Winter water was still present on the seafloor during late summer sampling. Close to shore, grain size at ICY2 to ICY5 was coarse (45% to 76% in phi 0-4 category), where *S. bradii* and *S. complanata* were predominant and *P. pseudopunctillata* was absent. The presence of *P. pseudopunctillata* beginning at ICY6 and steadily increasing to dominate (25% to 60%) the assemblages at ICY9, ICY10 and ICY11 is indicative of a seafloor sediment transition to very fine grains, ranging from 87% to 63% ≥5 phi. In addition to suitable habitat, persistent cold temperature supports *P. pseudopunctillata. Jonesia acuminata* is correlated to near-freezing temperatures and fine sediments (Gemery et al., 2013), and this species has a small but notable presence at the ICY7 to ICY11 stations.

Offshore Hanna Shoal, Northeast Chukchi Sea

Heterogeneous seafloor contours direct BSW with high nutrient concentrations to the Hanna Shoal area (Weingartner et al., 2017b). It is a hotspot for benthic productivity that supports marine mammals (Grebmeier et al., 2015a). The ostracode biofacies includes *P. pseudopunctillata* (37% of assemblage) with secondary species *S. complanata* (14%), *H. fascis* (8%), *R. tuberculatus* (7%) and *C. cluthae* (5%; Fig 11). It is very similar to the assemblage of offshore Icy Cape sites except this biofacies includes greater proportions of *A. dunelmensis* (6%) and *R. tuberculatus*, and *Argilloecia* sp. (4%), which are species associated with finer-grained sediments (Fig 6b). *Normanicythere leioderma* is rare (1%). *Paracyprideis pseudopunctillata* and *H. fascis* are euryhaline and can tolerate fluctuating salinities. At the time of summer collection, the average salinity at sample locations was 33, bottom temperature, -1.7°C and water depth, 51m. Sea ice tends to be trapped over Hanna Shoal, so dense bottom-confined pools formed by winter water tend to stagnate (Martin and Drucker, 1997;

Weingartner et al., 2005, 2013). Sustained cold temperatures, fine-grained sediments, and relatively higher salinities describe this frigid-water assemblage.

Barrow Canyon/DBO5

Sampling stations on the DBO5 transect have significant changes in depth, from ~50m at DBO 5-1 and DBO5-2, ~90m at DBO5-3 and deepening further to ~130m at DBO5-5, ~120m at DBO5-6, ~90m at DBO5-7 and ascending to 60-70m depths at DBO5-8 to DBO5-10 (Fig 2). Although seasonally variable, the deeper locations had much higher sediment chlorophyll-a (18-26 mean mg/m^2) values measured in summer, deposited by variable strong currents flowing through the Canyon. *N. leioderma* increases in abundance at these sites, particularly DBO5-6 and 5-7 stations, which historically also have high TOC values (~2%; Fig 12). These offshore stations are usually characterized by bottom water temperatures <0°C, however nearshore DBO5-1 and 5-2 sites have experienced summer warming in recent years with temperatures reaching 4-7°C (Fig 4d). Station DBO5-1 is influenced by ACW, which can reach the seafloor. These nearshore stations are dominated by *S. bradii* and cold-temperate/subarctic taxa *M. mananensis* and *S. ikeyai*, which decline in abundance moving seaward towards DBO5-5. (Note: Sites DBO5-4 and 5-8 are not represented due to low ostracode abundance of <30 specimens/sample.) At stations DBO5-6 to 5-10, seafloor sediments are extremely fine-grained silt, typically ≥90% 5phi. These sediments are the finest-grained of any ecoregion, and they are occupied by ostracode faunas that require this habitat along with near-freezing temperatures, notably *P. pseudopunctillata, S. complanata* and *C. cluthae*. At station DBO5-9 there is a distinct faunal transition to these species. Cold (<0°C), dense (~33-34 salinity) WW can remain at these stations during summer (Pickart et al., 2011). Occasionally, warm (>-1.2°C), salty (>33.6) Atlantic water is upwelled into the Canyon and fills the deepest part of the area (Pickart et al., 2019).

Alaskan Beaufort Sea

The Alaskan Beaufort continental shelf is a highly variable environment, influenced by seasonal discharge from the Colville and Mackenzie rivers that supply fresh water and deposit terrigenous material (Fig 3). Bottom water salinity at the sampling sites during the time of collection averaged 31 (sample locations were limited to within 100 km of shore, 20-90m water depth, and included intermittent years between 1971 and 2018). In addition to river inflow, the Beaufort shelf also receives dense ‘‘winter-transformed’’ Pacific water (Weingartner et al., 1998; Pickart, 2004) that drains off the Chukchi shelf. This water enters the Beaufort Sea as a shelf-break current that turns eastward from

Barrow (Fig 2; Pickart et al., 2005). As an area that experiences dramatic seasonal fluctuations, particularly in salinity, two species are overwhelmingly suited to these conditions: *P. pseudopunctillata* and *H. sorbyana*, cryophilic taxa correlated with euryhaline conditions (Fig 13; Stepanova et al., 2003, 2007). These two species are reported by Stepanova et al. (2003, 2007) as adapted to the inner shelf, river-affected zone of the Kara and Laptev seas where salinities range between 26 and 32. The eastern Alaskan Beaufort is influenced by the Mackenzie River, resulting in finer sediment grain sizes and better sorting in that region (Naidu, 1974; Barnes et al.,1982), which correlates to greater abundance of *P. pseudopunctilla*. For 2018 PRW and PRB transect samples (sites on Fig 3 map) that are most influenced by Colville River inflow, *H. sorbyana* is more prevalent than *P. pseudopunctillata*. Secondary species in the Beaufort Sea assemblage include *S. bradii, S. punctillata, R. tuberculatus, H. fascis, and A. dunelmensis. S. complanata* is present in low (~2%) abundance in these Beaufort Sea assemblages.

Despite limited temporal data in this area, *N. leioderma* and *S. bradii* are more prevalent in 2018 samples than in samples collected in comparable locations during the 1970s and 1980s where *P. pseudopunctillata* and *H. sorbyana* comprised the major proportion of the assemblage (S3 Fig). This may be due to recent declines in ice persistence and increases in nearshore temperatures or changes in sediment composition that may be incompatible with *P. pseudopunctillata's* preferences for near-freezing temperatures and finer grained sediments. However, sediment transport by ice rafting can be significant on the shelf, and can carry gravel and sand particles from the coast onto the shelf (Barnes et al., 1982). There are insufficient data available to determine if a change in sediment grain size is a factor. The Beaufort shelf, while not as productive as the Chukchi Sea, can have locally high primary production rates associated with the ice edge, reaching 200mg C m^{-2} d^{-1} (Hill et al., 2018). The retreating sea-ice edge, in addition to upwelling, provides nutrients for phytoplankton growth. The number and strength of upwelling events in the Alaskan Beaufort Sea has increased over the past 25 years (Pickart et al., 2013a, 2013b). Greater fresh phytoplankton production may be an explanation for the increasing appearance of *N. leioderma* in 2018 samples (S3 Fig), but our new data must be considered cautiously given the single year of samples.

Temporal trends in BCB Sea indicator species

Criteria used to assess whether environmental factors are driving changes in benthic communities include the arrival of taxa characteristic of a temperate climate zone or shifting dominance or abundance of taxa (Bluhm et al., 2017). Species can extend north and south (and east to west) to the point where certain maximum or minimum tolerable temperatures are reached (Hazel, 1970).

Due to temporal and spatial gaps in sampling, we could confidently evaluate temporal changes only in the dominant species, *N. leioderma, S. bradii* and *P. pseudopunctillata*. Two PCA comparisons by years (1970-2012 and 2013-2018) showed that the dominant species have remained fairly consistent in the BCB Seas (Fig 14). Interannual abundances of the dominant species in the Chukchi Sea from 2000-2018 did not reveal statistically significant changes, but two indicator species, *S. ikeyai* and *M. mananensis,* show incipient increases (Fig 15).

Dispersal of meiofauna into new areas likely lags behind sediment and water column changes. Benthic marine ostracodes are transported by ocean currents (Teeter, 1973). Considering that the movement of Pacific water from the Aleutian Passes to Bering Strait takes more than a year (Wood et al., 2015a) and an additional ~4 months to transit to Barrow Canyon in the Chukchi Sea (Woodgate et al., 2005a), benthic meiofaunal migration from the north Pacific to the Arctic could take a number of years. Over the shallow shelves of the northern Bering and Chukchi Seas, seasonal ice coverage cools the entire water column to temperatures below 0°C. These cold temperatures limit the northern distribution of subarctic populations of groundfish (Mueter and Litzow, 2008) and may also serve as a barrier inhibiting the mixing of Arctic and N. Pacific faunas through the Bering Strait (Joy and Clark, 1977). It is more likely that cold temperate species already inhabiting restricted inner bay areas like Norton Sound and coastal polynyas along Alaska will increasingly be able to extend their range and abundance as conditions become more suitable for them under changing climate scenarios.

Conclusions

We examined benthic ostracode assemblages collected during research cruises from 1970-2018 in the northern Bering, Chukchi and Alaskan Beaufort Seas. We focused on identifying indicator species and their associated ecological preferences. In particular, ostracode abundance and distribution were related to bottom temperature, salinity, organic carbon deposition (sedchla, TOC, C/N), and sediment substrate.

The variability in ostracode assemblage composition throughout the BCB Seas was linked qualitatively to localized (summer seasonal) environmental patterns. We found south-to-north (Chirikov Basin to Northern Chukchi to Beaufort) changes in dominant ostracode species and assemblage composition. Over the large biogeographic scale, four ostracode biofacies were identified in the study area primarily tied to the transit of summer water masses. Distinct changes of ostracode biofacies in nearshore to offshore transects were related to water mass properties likely in combination with food sources and sediment substrate. This study is among the first to highlight

changes in ostracode abundance and species over small spatial scales in response to changes in water mass properties and productivity.

Six indicator species were identified by correspondence and multivariate analyses based upon their abundance and correlations with distinct ecological parameters by which they are defined:

Normanicythere leioderma – opportunistic subarctic species correlated with ample, high-quality production (exported phytoplankton) as food source; sandy to pebbly to gravelly sediments;

Paracyprideis pseudopunctillata – euryhaline cryophilic species associated with WW presence and sea ice, fine grained sediments, sustained frigid temperatures (≤1°C), TOC with high phytodetritus;

Sarsicytheridea bradii -- habitat generalist, eurytopic species that capitalizes in highly dynamic environments, higher frequency in sandy sediments;

Semicytherura complanata – frigid temperatures (≤1°C), normal marine salinity, no discernable sediment preference;

Schizocythere ikeyai – cold-temperate species, warmer temperatures, ACW, sandy sediments;

Munseyella mananensis – cold-temperate species, warmer temperatures, ACW, sandy sediments.

This study found a consistent link between areas of high sediment chlorophyll, coarse sediment grain size and the abundance of *N. leioderma*. Despite wide tolerances, it may be a sensitive indicator of new production reaching the benthos and/or enhanced food supply. In future paleoceanographic studies using fossil ostracode faunal assemblage data, *N. leioderma* may signal the influence of nutrient-rich Pacific water flowing in through the Bering Strait and a food supply consisting of newly settled phytoplankton. *Paracyprideis pseudopunctillata* is positively correlated to very fine-grained sediment textures with high TOC values of refractory (detrital) organic carbon food sources.

During the last decade, we document an incipient increase in two secondary cold-temperate species, *S. ikeyai* and *M. mananensis*, that may be increasing. Preliminary interpretation of this finding may reflect recent increases in coastal and mid-shelf bottom water temperatures and/or carbon flux to the benthos.

Continued monitoring of temperature-sensitive ostracode species in the BCB Seas is necessary to provide information on annual and decadal variability in species distributions. This analysis of modern species ecology can help interpretation of ostracode faunal data from sediment cores in

relation to past and future ocean changes. This study contributes new species ecology and further validates the sensitivity and application of ostracode fauna as biomonitors and proxies for specific environmental conditions. These results provide a baseline for assessing the effects of future water mass changes and productivity on benthic ostracode communities in the Pacific Arctic.

Data Availability

AOD-2020 is available at NOAA's National Centers for Environmental Information (NCEI) World Data Service for Paleoclimatology for download (https://www.ncdc.noaa.gov/paleo/study/32312; Cronin et al., 2021). Data obtained during the DBO sampling efforts are available at the Earth Observing Laboratory (EOL) of the National Center for Atmospheric Research (NCAR), http://www.eol.ucar.edu/field_projects/dbo and at the Arctic Data Center (https://arcticdata.io/catalog/portals/DBO), supported by the National Science Foundation.

Supporting Information

S1 Table. **Six ecologically defined species in the northern Bering, Chukchi, and Beaufort Seas based on multivariate correlations with environmental factors.** (*=from listed ecological references)

S1 Figure. a. **Faunal abundance of selected taxa in relation to near-bottom temperature during summer sediment collection in the northern Bering and Chukchi Seas**. (n=211, 26,170 total specimens, 1990-2018, ≥30 specimens/sample).

b. **Faunal abundance in relation to salinity.**

c. **Faunal abundance in relation to sediment type.** Ostracode species abundance plotted against the percent sediment modal grain size of phi 0-4, where 0 represents gravel and rocks, 1 = coarse sand, 2 = medium sand, 3-4 = finer sand. Phi ≥5 (not shown) represents the very fine silty mud and clay sediment fraction typical of offshore or interior areas of the continental shelf.

S2 Figure. **Faunal abundance plotted against three different sources of sediment carbon, which may suggest preferred food sources**: a. sediment chlorophyll-a (sedchla), b. total organic carbon (TOC) and c. carbon to nitrogen (C/N) ratios.

S3 Figure. **Principal correspondence analysis (PCA) of Alaskan Beaufort Sea ostracode assemblages.** Sample sites are designated by collection years (legend symbols) and major taxa

(green lines with species names labeled in blue). The dominant species in the Alaskan Beaufort Sea are *H. sorbyana, P. pseudopunctillata, S. bradii* and, in samples collected in 2018, *N. leioderma*.

Acknowledgements

We thank A. Ruefer, S. Watson and N. Vaka for assistance with sample processing and E. Fachon for collection of 2018 Beaufort Sea samples during the HLY18-03 expedition. We also thank the field technicians in the Grebmeier/Cooper laboratory at CBL/UMCES for water column and sediment collections for multiple time series cruises for this study. We would also like to thank J. Keith, M. Robinson, G. L. Wingard, and two anonymous reviewers for critical comments that improved earlier versions of the manuscript. Any use of trade, firm, or product names is for descriptive purposes only and does not imply endorsement by the U.S. Government. Financial support for sample collections was provided by grants to JMG and LWC from the NSF Arctic Observing Network program (1204082, 1702456 and 1917469), NOAA Arctic Research Program (CINAR 22309.07 and 25984.02, https://arctic.noaa.gov/), PacMARS (Pacific Marine Arctic Regional Synthesis) project funded by Shell Exploration and Production and ConocoPhillips, and administered and managed by the North Pacific Marine Research Institute (NPMRI Arctic Project A01) through the North Pacific Research Board, and with oversight from the National Science Foundation (NSF) Division of Polar Programs, and BOEM funding through the COMIDA Hanna Shoal Project (UTA11-000872); and the USGS Climate & Land Use R&D Program.

Author Contributions

Conceptualization: L. Gemery
Data Curation: L. Gemery, T. M. Cronin
Formal analysis: L. Gemery
Funding acquisition: L. Gemery, L. W. Cooper, J. M. Grebmeier
Investigation: L. Gemery, L. W. Cooper, J. M. Grebmeier
Methodology: L. Gemery
Supervision: L. W. Cooper, T. M. Cronin
Visualization: L. Gemery
Writing – original draft: L. Gemery
Writing – review & editing: L. Gemery, T. M. Cronin, L. W. Cooper, H. J. Dowsett, J. M. Grebmeier

References

arcticdata.io [Internet]. NSF, Arctic Data Center. Available from [https://arcticdata.io/catalog/portals/DBO/].

Arrigo R, van Dijken GL. Continued increases in Arctic Ocean primary productions. Progress in Oceanography. 2015; 136: 60-70. doi:10.1016/j.pocean.2015.05.002.

Barnes PW, Reimnitz E, Fox D. Ice rafting of fine-grained sediment, a sorting and transport mechanism, Beaufort Sea, Alaska. Journal of Sedimentary Petrology. 1982; 52 (2): 493-502.

Blanchard AL, Parris CL, Knowlton AL, Wade NR. Benthic ecology of the northeastern Chukchi Sea. Part I: Environmental characteristics and macrofaunal community structure, 2008-1010. Continental Shelf Research. 2013; 67: 52-66. doi:10.1016/j.csr.2013.04.021.

Bluhm BA, Swadling KM, Gradinger R. Sea ice as a habitat for macrograzers. In: Thomas DN (Ed.). Sea Ice. United Kingdom: John Wiley & Sons Ltd. 2017; 3: 394-414. doi:10.1002/9781118778371.ch16.

Boyer TP, Stephens C, Antonov JI, Conkright ME, Locarnini, RA, O'Brien TD, et al. World Ocean Atlas 2001, Volume 2: Salinity [CD-ROM]. Levitus S (Ed.), NOAA Atlas NESDIS 50, U.S. Government Printing Office, Washington D.C. 2002; 165.

Boyer TP, Antonov JI, Baranova OK, Coleman C, Garcia HE, Grodsky A, et al. World Ocean Database, NOAA Atlas NESDIS 72. Levitus S (Ed.), Mishonov A (Technical Ed.), Silver Spring, MD. 2013; 209. doi:10.7289/V5NZ85MT. Available from [https://www.nodc.noaa.gov/OC5/WOD13/].

Brouwers EM. Pliocene Paleoecologic Reconstructions based on Ostracode Assemblages from the Sagavanirktok and Gubik Formations, Alaskan North Slope. U.S. Geological Survey Professional Paper. 1992; OFR 92-321.

Brown ZW, van Dijken GL, Arrigo KR. A reassessment of primary production and environmental change in the Bering Sea. Journal of Geophysical Research. 2011; 116: C08014.

Brown ZW, Arrigo KR. Contrasting trends in sea ice and primary production in the Bering Sea and Arctic Ocean. ICES Journal of Marine Science. 2012; 69: 7, 1180-1193. doi:10.1093/icesjms/fss113.

Coachman LK, Aagaard K, Tripp RB. Bering Strait: The Regional Physical Oceanography. University of Washington Press, Seattle. 1975.

Cooper LW, Grebmeier JM, Larsen IL, Egorov VG, Theodorakis C, Kelly HP, et al. Seasonal variation in sedimentation of organic materials in the St. Lawrence Island polynya region, Bering Sea. Marine Ecology Progress Series. 2002; 226: 13-26.

Cooper LW, Savvichev AS, Gremeier JM. Abundance and production rates of heterotrophic bacterioplankton in the context of sediment and water column processes in the Chukchi Sea. Oceanography. 2015; 28 (3): 84-99. doi:10.5670/oceanog.2015.59.

Cooper LW, Grebmeier JM. Deposition patterns on the Chukchi shelf using radionuclide inventories in relation to surface sediment characteristics. Deep Sea Research Part II: Topical Studies in Oceanography. 2018; 152: 48-66. doi:10.1016/j.dsr2.2018.01.009.

Cronin TM. Champlain Sea foraminifera and ostracoda: A systematic and Paleoecological synthesis. Géographie physique et Quaternaire. 1977; 31: 107-122.

Cronin TM, Briggs Jr. WM, Brouwers EM, Whatley RC, Wood A, Cotton MA. Modern Arctic podocopid ostracode database. United States Geological Survey Open-File Report. 1991; 91-385: 1-51.

Cronin TM, Gemery L, Brouwers EM, Briggs Jr. WM, Wood A, Stepanova A, et al. Modern Arctic ostracode database. IGBP PAGES/WDCA. 2010; Contribution Series Number: 2010-08. Available from [ftp://ftp.ncdc.noaa.gov/pub/data/paleo/contributions_by_author/cronin2010/cronin2010.txt.].

Cronin TM, Gemery L., Briggs, Jr. WM, Brouwers EM, Schornikov EI, Stepanova A, Wood A, Yasuhara M, Siu S. Arctic Ostracode Database 2020. NOAA's National Centers for Environmental Information (NCEI). 2021. Available from [https://www.ncdc.noaa.gov/paleo/study/32312].

Danielson SL, Eisner L, Ladd C, Mordy C, Sousa L, Weingartner TJ. A comparison between late summer 2012 and 2013 water masses, macronutrients, and phytoplankton standing crops in the northern Bering and Chukchi Seas. Deep-Sea Research Part II. 2017; 135: 7-26. doi:10.1016/j.dsr2.2016.05.024.

Danielson SL, Ahkinga O, Ashjian C, Basyuk E, Cooper LW, Eisner L, et al. Manifestation and consequences of warming and altered heat fluxes over the Bering and Chukchi Sea continental shelves. Deep Sea Research Part II: Topical Studies in Oceanography. 2020; 177: 104781. doi:10.1016/j.dsr2.2020.104781.

Elofson O. Zur Kenntnis der marinen Ostracoden Schwedens mit besonderer Berucksichtigung des Skageraks. Zoologiska Bidrag fran Uppsala. (Marine Ostracoda of Sweden with special consideration of the Skagerrak, Translation from German into English, 1969. Israel Program for Scientific Translations: 286 pp.). 1941; 19: 215-534, Figures 1-52.

Feder HM, Naidu AS, Jewett SC, Hameedi JM, Johnson WR, Whitledge TE. The northeastern Chukchi Sea: benthos-environmental interactions. Marine Ecology Progress Series. 1994; 111 (1/2): 171-190.

Frenzel P, Boomer I. The use of ostracods frim. Arginal marine, brackish waters as bioindicators of modern and quaternary environmental change. Palaeogeography, Palaeoclimatology, Palaeoecology. 2005; 225: 68-92.

Frey KE, Moore GWK, Cooper LW, Grebmeier JM. Divergent patterns of recent sea ice cover across the Bering, Chukchi and Beaufort seas of the Pacific Arctic Region. Progress in Oceanography. 2015; 136: 32-49. doi:10.1016/j.pocean.2015.05.009.

Frey KE, Comiso JC, Cooper LW, Grebmeier JM, Stock LV. Arctic ocean primary productivity: The response of marine algae to climate warming and sea ice decline. In: Arctic Report Card, NOAA. 2020. Available from [https://arctic.noaa.gov/Report-Card/Report-Card-2020/ArtMID/7975/ArticleID/900/Arctic-Ocean-Primary-Productivity-The-Response-of-Marine-Algae-to-Climate-Warming-and-Sea-Ice-Decline]. doi:0.25923/vtdn-2198

Gemery L, Cronin TM, Cooper LW, Grebmeier JM. Temporal changes in benthic ostracode assemblages in the Northern Bering and Chukchi Seas from 1976 to 2010. Deep-Sea Research II: Topical Studies in Oceanography. 2013; 94: 68-79.

Gemery L, Cronin TM, Briggs WM, Brouwers EM, Schornikov E, Stepanova A, et al. An arctic and subarctic ostracode database: Biogeographic and Paleoceanographic applications. Hydrobiologia. 2015; 786 (1): 59-95. doi:10.1007/s10750-015-2587-4.

Gemery L, Cronin TM, Poirier RK, Pearce C, Barrientos N, O'Regan M, et al. Central Arctic Ocean paleoceanography from ~50 ka to present, on the basis of ostracode faunal assemblages from SWERUS 2014 expedition. Climate of the Past. 2017. doi.org/10.5194/cp-2017-22, 2017.

Gong D, Pickart RS. Summertime circulation in the eastern Chukchi Sea. Deep Sea Research Part II: Topical Studies in Oceanography. 2015; 118 (Part A): 18-21. doi:10.1016/j.dsr2.2015.02.006

Grebmeier JM, Overland JE, Moore SE, Farley EV, Carmack EC, Cooper LW, et al. A major ecosystem shift in the Northern Bering Sea. Science. 2006b; 311 (5766): 1461-1464.

Grebmeier JM. Shifting patterns of life in the Pacific Arctic and sub-Arctic seas. Annual Review of Marine Science. 2012; 4: 63-78. doi:10.1146/annurev-marine-120710-100926.

Grebmeier JM, Cooper LW. Bottom Water Temperature and Salinity (2000-2012). PacMARS. 2014a; Version 1.0. Available from [PacMARS EOL data archive site http://pacmars.eol.ucar.edu]. doi:10.5065/D6VM49BM.

Grebmeier J, Cooper LW. PacMARS sediment chlorophyll, a. Version 1.0., UCAR/NCAR. Earth Observing Laboratory. 2014b; cited [2020 August 26]. doi:10.5065/D6W9576K.

Grebmeier JM, Bluhm BA, Cooper LW, Danielson S, Arrigo KR, Blanchard AL, et al. Ecosystem characteristics and processes facilitating persistent macrobenthic biomass hotspots and associated benthivory in the Pacific Arctic. Progress in Oceanography. 2015a; 136: 92-114.

Grebmeier JM, Bluhm BA, Cooper LW, Denisenko SG, Iken K, Kedra M, et al. Time-series benthic community composition and biomass and associated environmental characteristics in the Chukchi Sea during the RUSALCA 2004-2012 Program. Oceanography. 2015b; 28: 116-133. doi:10.5670/oceanog.2015.61.

Grebmeier J, Cooper L. PacMARS Surface Sediment Parameters (1970-2012). Version 2.0., UCAR/NCAR - Earth Observing Laboratory. 2016; cited [2020 August 24]. doi:10.5065/D6416V3G. Data provided by NCAR/EOL under the sponsorship of the National Science Foundation. Available from [https://data.eol.ucar.edu/].

Grebmeier JM, Frey KE, Cooper LW, Kedra M. Trends in benthic macrofaunal populations, seasonal sea ice persistence, and bottom water temperatures in the Bering Strait region. Oceanography. 2018; 31(2): 136-151. doi:10.5670/oceanog.2018.224.

Hammer Ø, Harper DAT, Ryan PD. PAST: Paleontological statistics software package for education and data analysis. Palaeontologia Electronica. 2001; 4 (1): 9. Available from [https://past.en.lo4d.com/windows].

Hazel JE, Valentine PC. Three new ostracods from off northeast North America. Journal of Paleontology. 1969; 43: 741-752.

Hazel JE. Ostracode zoogeography, Nova Scotian and Virginian faunal provinces. Geological Survey Professional Paper. 1970; 529-E.

Hill V, Ardyna M, Lee SH, Varela DE. Decadal trends in phytoplankton production in the Pacific Arctic Region from 1950 to 2012. Deep Sea Research Part II. 2018; 152: 82-94. doi:10.1016/j.dsr2.2016.12.015.

Horne D, Holmes J, Viehberg F, Rodriquez-Lazaro J. Ostracoda as proxies for Quaternary climate change. In: Jaap JM and van derMeer JJM (Eds.) Developments in Quaternary Science, Volume 17, Elsevier, Amsterdam. 2012; 1-337.

Huntington HP, Danielson SL, Wiese FK, Baker M, Boveng P, Citta JJ, et al. Evidence suggests potential transformation of the Pacific Arctic ecosystem is underway. Nature Climate Change. 2020; 10: 342-348. doi:10.1038/s41558-020-0695-2.

Hutchins LW. The bases for temperature zonation in geographical distribution. Ecological Monographs. 1947; 17: 325-335.

Ikeya N, Cronin TM. Quantitative analysis of ostracoda and water masses around Japan: Application to Pliocene and Pleistocene paleoceanography. Micropaleontology. 1993; 39 (3): 263-281.

Joy JA, Clark DL. The distribution, ecology and systematics of the benthic ostracoda of the central Arctic Ocean. Micropaleontology. 1977; 23: 129-154.

Kornicker LS. Distribution of the ostracode suborder Cladocopa, and a new species from the Bahamas. Micropaleotology. 1959; 5 (1): 69-75.

Kornicker LS, Wise CD. Some environmental boundaries of a marine ostracode. Micropaleontology. 1960; 6 (4): 393-398.

Legendre L and Legendre P. Numerical Ecology, Developments in Environmental Modelling. Elsevier Scientific Publ. Co., Amsterdam, Netherlands, Second English Edition. 1998; 870. ISBN: 0-444-89249-4.

Lewis KM, van Dijken GL, Arrigo KR. Changes in phytoplankton concentration now drive increased Arctic Ocean primary production. Science. 2020; 369 (6500): 198-202. doi:10.1126/science.aay8380.

Martin S, Drucker R. The effect of possible Taylor columns on the summer ice retreat in the Chukchi Sea. Journal of Geophysical Research: Oceans. 1997; 102 (C5). doi:10.1029/97JC00145.

Millero FJ. What is PSU? Oceanography. 1993; 6 (3): 67.

Moore SE and Grebmeier JM. The distributed biological observatory: Linking physics to biology in the Pacific Arctic region. Arctic. 2018; 71: 1–7.

Mueter FJ, Litzow MA. Sea ice retreat alters the biogeography of the Bering Sea continental shelf. Ecological Applications. 2008; 18 (2): 309-320.

Naidu AS. Sedimentation in the Beaufort Sea: A synthesis. Marine Geology and Oceanography of the Arctic Seas. 1974; 173-190.

Neale JW, Howe HV. The marine ostracoda of Russian Harbor, Novaya Zemlya and other high latitude faunas. Bulletin of American Paleontology. 1975; 65: 282, 381-431.

Nelson RJ, Ashjian C, Bluhm B, Conlan K, Gradinger R, Grebmeier J, et al. Biodiversity and biogeography of the lower trophic taxa of the Pacific Arctic region: sensitivities to climate change. In: Grebmeier JM, Maslowski W (Eds.). The Pacific Arctic Region: Ecosystem Status and Trends in a Rapidly Changing Environment. Dordrecht: Springer. 2014; 269-336.

Okkonen, S. CTD Summary Data. Version 1.0. UCAR/NCAR - Earth Observing Laboratory. 2013; cited [2020 December 15]. doi:10.5065/D6DB7ZVJ. Data provided by NCAR/EOL under the sponsorship of the National Science Foundation. Available from [https://data.eol.ucar.edu/].

Ozawa H. Japan Sea ostracod assemblages in surface sediments: their distribution and relationships to water mass properties. Paleontological Research. 2003; 7 (3): 257-274.

Ozawa H. Okhotsk Sea ostracods in surface sediments: Depth distribution on cryophilic species relative to oceanic environment. Marine Micropaleontology. 2004; 53: 245-260. doi:10.1016/j.marmicro.2004.06.002.

pacmars.eol.ucar.edu [Internet]. Pacific Marine Arctic Regional Synthesis Data Archive; c2012. Available from [http://pacmars.eol.ucar.edu/].

Pickart RS. Shelfbreak circulation in the Alaskan Beaufort Sea: Mean structure and variability. Journal of Geophysical Research. 2004; 109: C04024. doi:10.1029/2003JC001912.

Pickart RS, Weingartner TJ, Pratt LJ, Zimmermann S, Torres DJ. Flow of winter-transformed Pacific watter into the Western Arctic. Deep Sea Research Part II: Topical Studies in Oceanography. 2005; 52 (24-26): 3175-3198. doi:10.1016/j.dsr2.2005.10.009.

Pickart RS, Moore GWK, Torres DJ, Fratantoni PS, Goldsmith RA, Yang J. Upwelling on the continental slope of the Alaskan Beaufort Sea: Storms, ice, and oceanographic response. Journal of Geophysical Research. 2009; 114: C00A13. doi:10.1029/2008JC005009.

Pickart RS, Spall MA, Moork GW, Weingartner TJ, Woodgate RA, Aagaard K, et al. Upwelling in the Alaskan Beaufort Sea: Atmospheric forcing and local versus non-local response. Progress in Oceanography. 2011; 88 (1-4): 78-100.

Pickart RS, Schulze LM, Moore GWK, Charette MA, Arrigo KR, van Dijken G, et al. Long-term trends of upwelling and impacts on primary productivity in the Alaskan Beaufort Sea. Deep-Sea Research I. 2013a; 79, 106-121.

Pickart RS, Spall MA, Mathis JT. Dynamics of upwelling in the Alaskan Beaufort Sea and associated shelf-basin fluxes. Deep-Sea Research. 2013b; 176: 35-51.

Pickart RS, Nobre C, Lin P, Arrigo KR, Ashjian CJ, Berchok C, et al. Seasonal to mesoscale variability of water masses and atmospheric conditions in Barrow Canyon, Chukchi Sea. Deep-Sea Research Part II. 2019. doi:10.1016/j.dsr2.2019.02.003.

Schornikov EI. Class ostracoda, orders Platycopida and Podocopida. In: Sirenko B.I. (Ed.). List of species of free-living invertebrates of Eurasian Arctic seas and adjacent deep waters: Explorations of the fauna of the seas. St. Peterburg, T. 2001; 51 (59): 99–103.

Serreze MC, Crawford AD, Stroeve JC, Barrett AP, Woodgate RA. Variability, trends, and predictability of seasonal sea ice retreat and advance in the Chukchi Sea. Journal of Geophysical Research: Oceans. 2016; 121: 7308-7325. doi:10.1002/ 2016JC011977.

Siddiqui QA, Grigg UM. A preliminary survey of the ostracodes of Halifax Inlet. In: Swaine FH (Ed.). Biology and paleobiology of ostracoda. Bulletin of American Paleontology. 1975; 65: 282, 369-379.

Sigler MF, Mueter FJ, Bluhm BA, Busby MS, Cokelet ED, Danielson S, et al. Late summer zoogeography of the northern Bering and Chukchi seas. Deep-Sea Research Part II. 2017; 135: 168-189. doi:10.1016/j.dsr2.2016.03.005.

Stabeno PJ, Duffy-Anderson JT, Eisner LB, Farley EV, Heintz RA, Mordy CW. Return of warm conditions in the southeastern Bering Sea: Physics to fluorescence. PloS ONE. 2017; 12 (9): e0185464. doi:10.1371/journal.pone.0185464.

Stabeno PJ, Bell S, Bond NA, Kimmel D, Mordy C, Sullivan ME. Distributed Biological Observatory Region 1: Physics, chemistry and plankton in the northern Bering Sea. Deep-sea Research Part II: Topical Studies in Oceanography. 2019; 162: 8-21. doi:10.1016/j.dsr2.2018.11.006.

Stepanova A, Taldenkova E, Bauch HA. Recent ostracoda of the Laptev Sea (arctic Siberia): taxonomic composition and some environmental implications. Marine Micropaleontology. 2003; 48: 23-48.

Stepanova AY. Late Pleistocene-Holocene and recent ostracoda of the Laptev Sea. Monograph. Suppl. Issue Russ. Paleontological Journal. 2006; 40 (2): S91-S204.

Stepanova A, Taldenkova E, Simstich J, Bauch HA. Comparison study of the modern ostracod associations in the Kara and Laptev Seas: Ecological aspects. Marine Micropaleontology. 2007; 63 (3): 111-142.

Stephens C, Antonov JI, Boyer TP, Conkright ME, Locarnini RA, O'Brien TD, et al. World Ocean Atlas 2001, Volume 1: Temperature [CD-ROM]. Levitus S (Ed.), NOAA Atlas NESDIS 49, U.S. Government Printing Office, Washington D.C. 2002; 167.

Teeter JW. Geographical distribution and dispersal of some Recent shallow-water marine Ostracoda. Ohio Journal of Science, Akron. 1973; 73 (1): 46–54, 2 pls., 2 tabs.

Yasuhara M, Hunt, G, van Dijken G, Arrigo KR, Cronin TM, Wollenburg JE. Patterns and controlling factors of species diversity in the Arctic Ocean, Journal of Biogeography. 2012; 39, 2081–2088.

Walsh JJ, McRoy CP, Coachman LK, Goering JJ, Nihoul JJ, Whitledge TE, et al. Carbon and nitrogen cycling within the Bering Chukchi Seas – source regions for organic-matter effecting AOU demands of the Arctic Ocean. Progress in Oceanography. 1989; 22: 277-359. doi:10.1016/0079-6611(89)90006-2.

Wassmann P, Duarte CM, Agusti S, Sejr MK. Footprints of climate change in the Arctic marine ecosystem. Biological Global Change. 2011; 17 (2): 1235-1249. doi:10.1007/s00300-010-0839-3.

Weingartner TJ, Cavalieri DJ, Aagaard K, Sasaki Y. Circulation, dense water formation, and outflow on the northeast Chukchi shelf. Journal of Geophysical Research. 1998; 103: 7647-7661. doi:10.1029/98JC00374.

Weingartner TJ, Aagaard K, Woodgate R, Danielson S, Sasaki S, Cavalieri DY. Circulation on the north central Chukchi Sea shelf. Deep-Sea Research Part II. 2005; 52 (24-26): 3150-3174. doi:10.1016/j.dsr2.2005.10.01.

Weingartner T, Dobbins E, Danielson S, Potter R, Statscewich H, Winsor P. Hydrographic variability over the northeastern Chukchi Sea shelf in summer-fall 2008-2010. Continental Shelf Research. 2013; 67: 5-22. doi:10.1016/j.csr. 2013.03.012.

Weingartner T, Fang Y-C, Winsor P, Dobbins E, Potter R, Statscewich H, et al. The summer hydrographic structure of the Hanna Shoal region on the northeastern Chukchi Sea shelf: 2011–2013. Deep Sea Research Part II: Topical Studies in Oceanography. 2017b; 144: 6-20. doi:10.1016/j.dsr2. 2017.08.006.

Wood KR, Bond NA, Danielson SL, Overland JE, Salo SA, Stabeno P, et al. A decade of environmental change in the Pacific Arctic region. Progress in Oceanography. 2015a; 136: 12-31. doi:10.1016/j.pocean.2015.05.005.

Wood K, Wang J, Salo S, Stabeno P. The climate of the Pacific Arctic during the first RUSALCA decade 2004-2013. Oceanography. 2015b; 28: 24-35. doi:10.5670/oceanog.2015.55.

Woodgate RA, Aagaard K, Weingartner TJ. Monthly temperature, salinity and transport variability of the Bering Strait throughflow. Geophysical Research Letter. 2005a; 32: L04601. doi:10.1029/2004GL021880.

Woodgate RA, Aagaard K, Weingartner TJ. A year in the physical oceanography of the Chukchi Sea: Moored measurements from autumn 1990-1991. Deep Sea Research Part II. 2005b; 52 (24-26): 3116-3149. doi:10.1016/j.dsr2.2005.10.016.

Woodgate RA, Stafford KM, Prahl FG. A synthesis of year-round interdisciplinary mooring measurements in the Bering Strat (1990-2014) and the RUSALCA years (2004-2011). Oceanography. 2015; 28: 46-67. doi:10.5670/oceanog.2015.57.

Woodgate RA. Increases in the Pacific inflow to the Arctic from 1990 to 2015, and insights into seasonal trends and driving mechanisms from year-round Bering Strait mooring data. Progress in Oceanography. 2018; 160: 124-154. doi:10.1016/j.pocean.2017.12.007.

Figures and tables

Click here to access/download;Figure;NFigures 1-14 and Tables For submit.docx

Biogeography and Ecology of Ostracoda in the U.S. northern Bering, Chukchi, and Alaskan Beaufort Seas

Fig 1

Locations of samples used for the biogeographical study. (n=289 sediment aliquots with ≥30 specimens per sediment sample, 34,369 total specimens, 1970-2018) The blue “DBO” areas denote Distributed Biological Observatory stations. (Geographic coordinates are provided in Table 1.) Note that some stations along established DBO transects are not represented due to low or no ostracode abundance (i.e. DBO3-6 to 3-8 and DBO4).

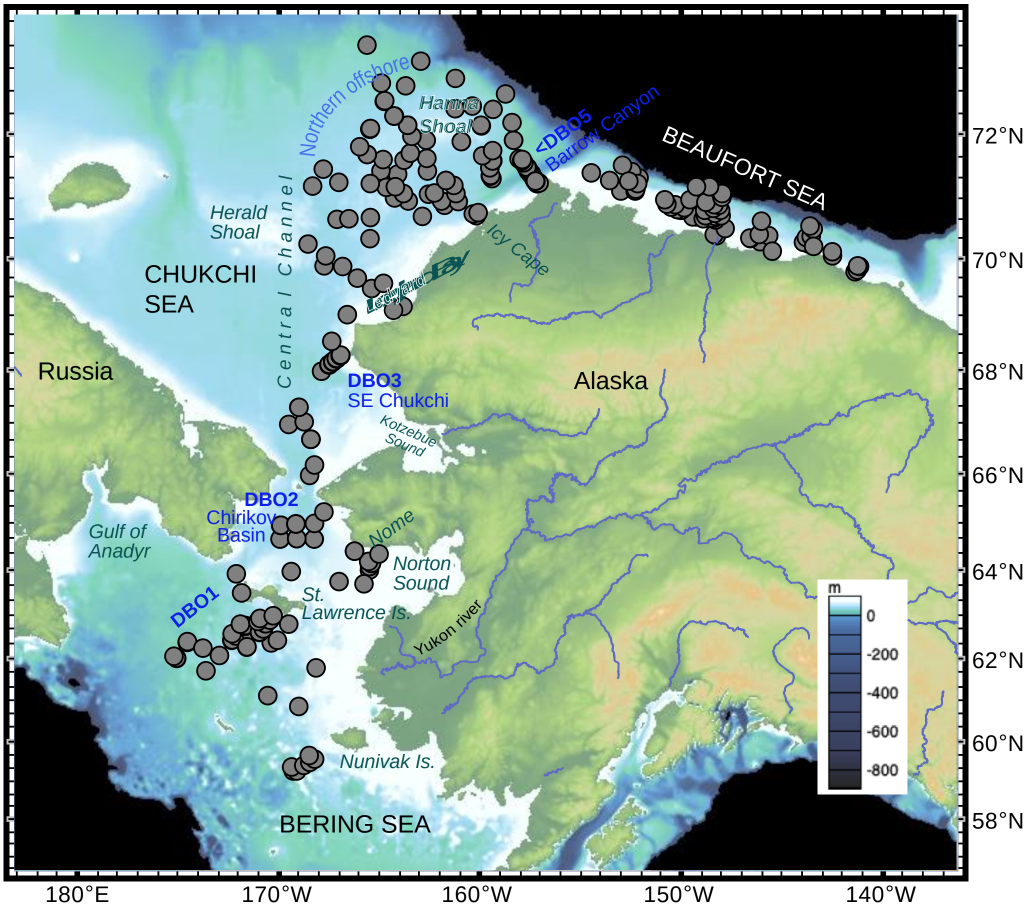

Fig 2

Location of sample sites in the northern Bering and Chukchi Seas used for ecoregion transect (nearshore to offshore) study. (n=147 surface sediment aliquots with ≥30 specimens per sediment sample, 19,300 total specimens, 1990-2018) Schematic of summertime surface current flow patterns and water mass type that can affect seafloor conditions (Anadyr Water [AW], Alaska Coastal Water [ACW], Bering Sea Water [BSW]; properties denoted in Table 3) adapted from Danielson et al. (2017, 2020).

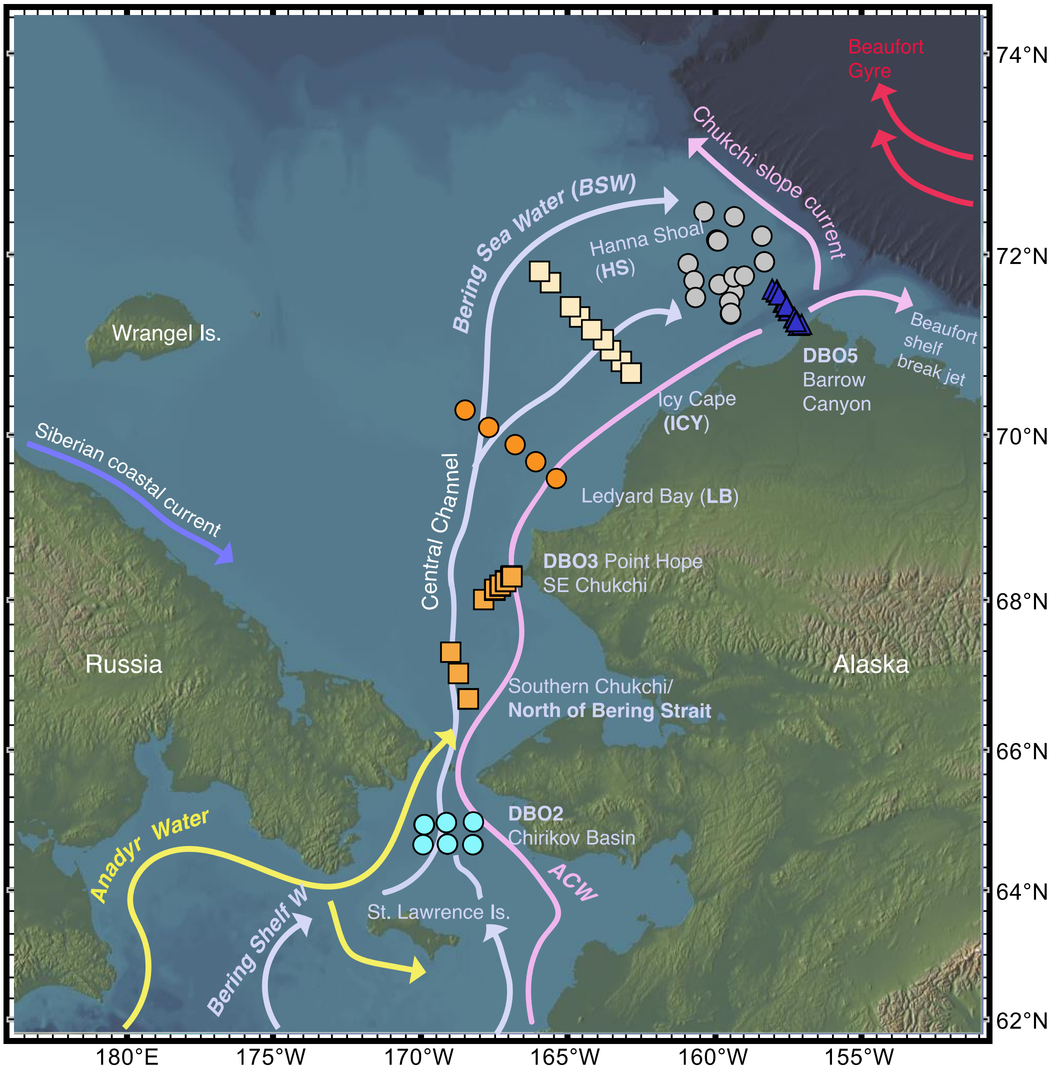

Fig 3

Location of samples used in Beaufort Sea ecological transect study from years 1971-2018.
(n=55 surface sediment aliquots with ≥30 specimens per sediment sample, 20-100mwd, 6,617 total specimens)

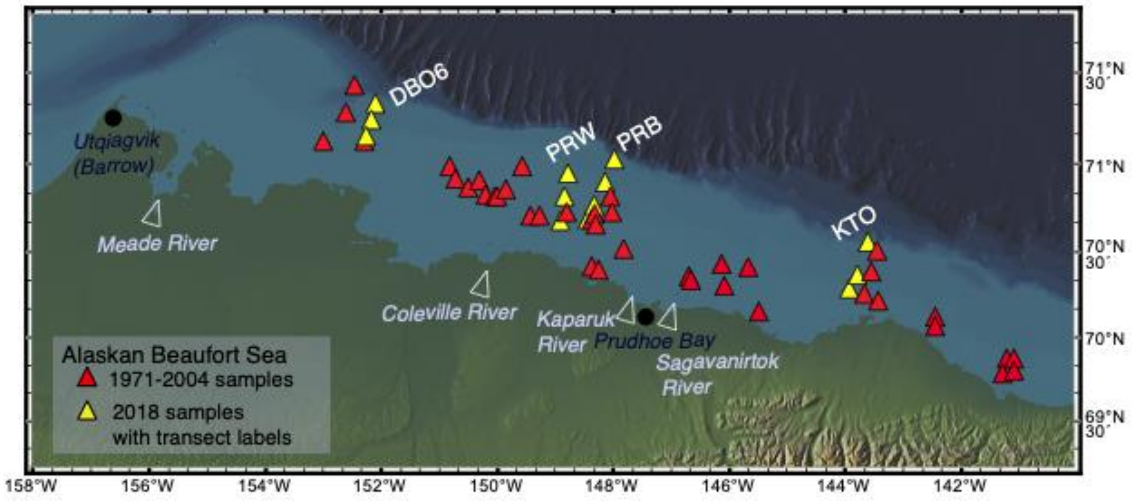

Fig 4

Summary bottom water temperature at ecoregions.

Comparison of summer bottom temperatures in studied ecoregions and months at stations nearest to shore (e.g. DBO3-1, DBO5-1) that are locations affected by Alaska Coastal Water, and more central or middle-shelf stations (DBO3-5 or DBO5-10) that are affected by Bering Sea Water (winter or summer variants). This subset of data was derived from the Pacific Marine Arctic Regional Synthesis (PacMARS) Project (Grebmeier and Cooper, 2014a; http://dx.doi.org/10.5065/D6VM49BM; Okkonen, 2013; https://data.eol.ucar.edu/dataset/10339) and recent expedition data (2014-2018) taken aboard USCGS Healy and CCGS Sir Wilfrid Laurier and archived at the National Science Foundation's Arctic Data Center.

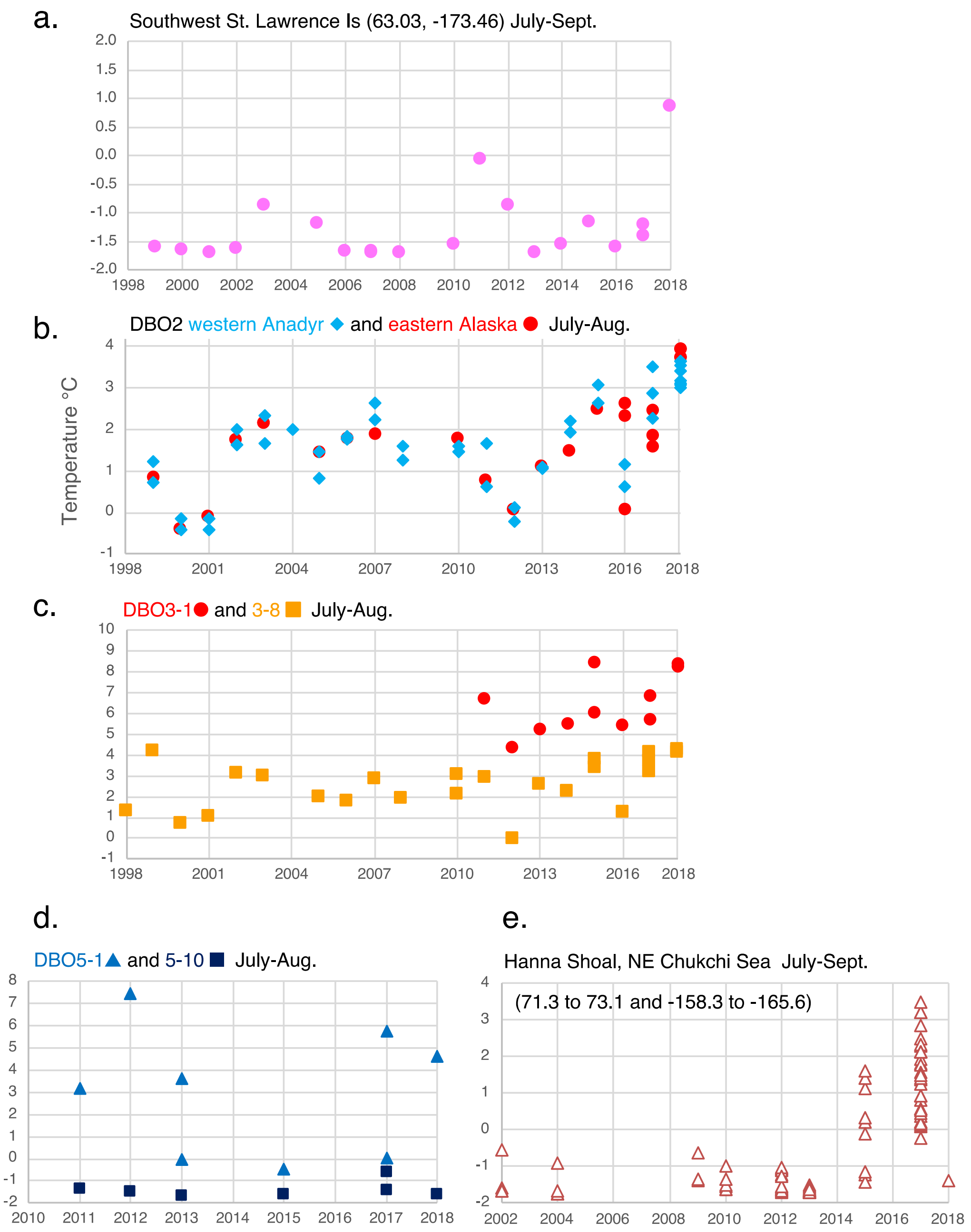

Fig 5

Principal correspondence analysis (PCA). PCA of ostracode assemblages in the northern Bering, Chukchi and Beaufort Sea region (locations on Fig.1; geographic coordinates in Table 1), with sites designated by DBO areas and other ecoregions (legend symbols) and major taxa (green lines with species names labeled in blue). Three species dominate the assemblage groups: *N. leioderma, S. bradii, and P. pseudopunctillata*. The proximity of the symbols in the plot reflects the level of similarity in taxa present at each sampling station.

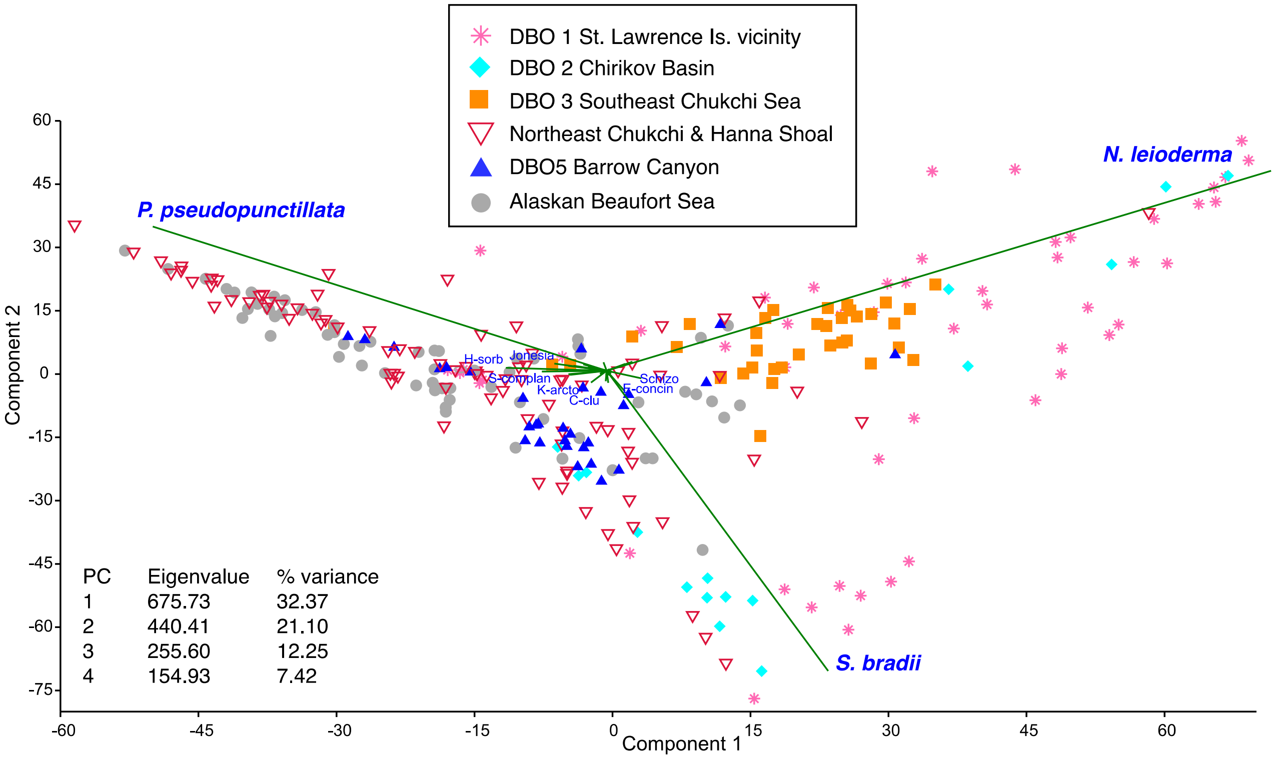

Fig 6

a. **Detrended correspondence analysis (DCA).** DCA using the northern Bering, Chukchi and Beaufort Seas biogeographic dataset (1970–2018), describes relationships between ostracode assemblage structure and geographic ecoregions in the region. Ostracode biofacies differed among ecoregions within the study area (described in Table 3) and grouped primarily on summer water mass characteristics (temperature and salinity).

b. **Canonical correspondence analysis (CCA).** CCA of ostracode assemblages in the northern Bering-Chukchi Sea region, with sampling ecoregion sites (noted by symbol on legend), major taxa (names are labeled in blue), and eight environmental parameters (green lines and labels in black: temperature, salinity, sediment total organic carbon [TOC], sediment nitrogen [TON], sediment chlorophyll-a [sedchla], phi ≥5 [fine silt], and phi 0-4 [gravel pebbles to sands]). Samples in the Hanna Shoal region (red dots) were separated from the larger northeastern Chukchi Sea sample set (inverted red triangle). Dataset includes the 14 most abundant species in 153 surface sediment aliquots (23,939 total specimens) from the northern Bering and Chukchi Seas during summers 1990-2018 for which sediment data was available.

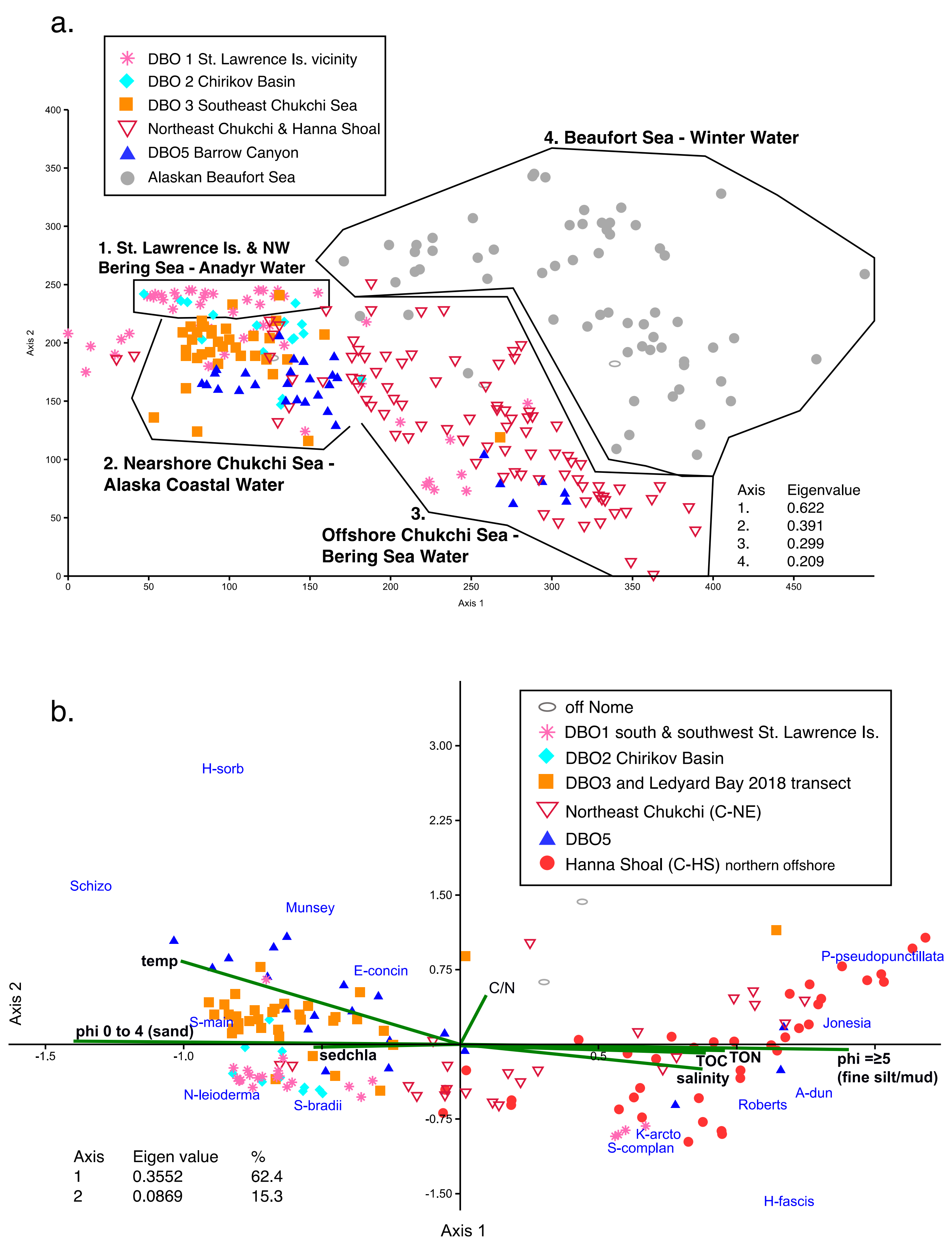
a.
DBO 1 St. Lawrence Is. vicinity
DBO 2 Chirikov Basin
DBO 3 Southeast Chukchi Sea
Northeast Chukchi & Hanna Shoal
DBO5 Barrow Canyon
Alaskan Beaufort Sea
1. St. Lawrence Is. & NW Bering Sea - Anadyr Water
2. Nearshore Chukchi Sea - Alaska Coastal Water
3. Offshore Chukchi Sea - Bering Sea Water
4. Beaufort Sea - Winter Water
Axis 2
Axis 1
0
50
100
150
200
250
300
350
400
450
Axis Eigenvalue
1. 0.622
2. 0.391
3. 0.299
4. 0.209
b.
off Nome
DBO1 south & southwest St. Lawrence Is.
DBO2 Chirikov Basin
DBO3 and Ledyard Bay 2018 transect
Northeast Chukchi (C-NE)
DBO5
Hanna Shoal (C-HS) northern offshore
H-sorb
Schizo
Munsey
E-concin
temp
C/N
S-main
phi 0 to 4 (sand)
sedchla
N-leioderma
S-bradii
TOC TON
salinity
phi =≥5 (fine silt/mud)
P-pseudopunctillata
Jonesia
A-dun
Roberts
K-arcto
S-complan
H-fascis
3.00
2.25
1.50
0.75
-0.75
-1.50
-1.5
-1.0
0.5
Axis 2
Axis 1
Axis Eigen value %
1 0.3552 62.4
2 0.0869 15.3

Fig 7

a. **Primary species, DBO2 - Chirikov Basin**. Stack plot of dominant species in DBO2- Chirikov Basin region (Fig. 2) in the northern Bering Sea (various years from 2001 to 2018; n=15, 1,314 specimens with ≥30 specimens/sample). The 11 species presented in the stack plot comprise 98% of the biofacies in this ecoregion. (Note the boundary between Anadyr water on the western side of Chirikov Basin and Bering Sea Water on the eastern side is generalized in this graphic.)

b. **Summer bottom water salinity at western and eastern DBO2 - Chirikov Basin sampling sites.** Time series plot of summer bottom salinity at sampling stations on the western and eastern side of the Chirikov Basin (Source: Grebmeier and Cooper, 2014a; Okkonen, 2013; and recent expedition data (2014-2018) taken aboard USCGS Healy and CCGS Sir Wilfrid Laurier archived at the Arctic Data Center)

a.

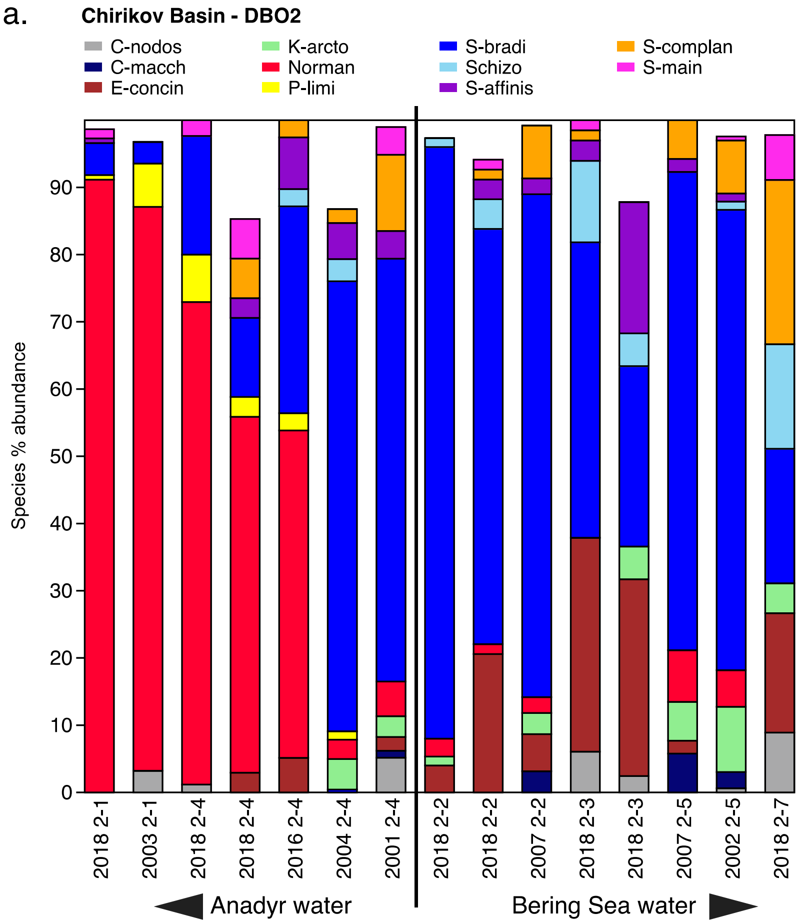

b.

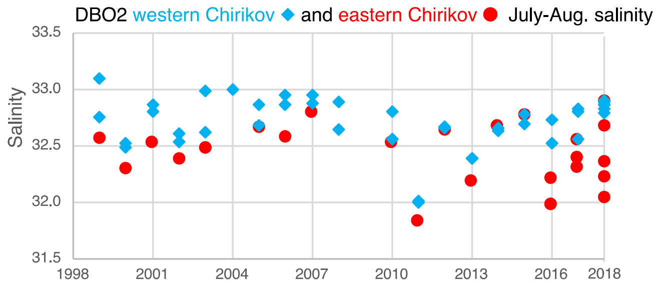

Fig 8

Primary species, DBO3 - Southern Chukchi Sea. Stack plot of the 12 most abundant species and one genus (*Semicytherura*), comprising 92% of the ostracode assemblage, in DBO3 region (sites on Fig. 2 map) from years ranging from 1998 to 2018, (n=32 samples with ≥30 specimens/sample, 7,514 total specimens).

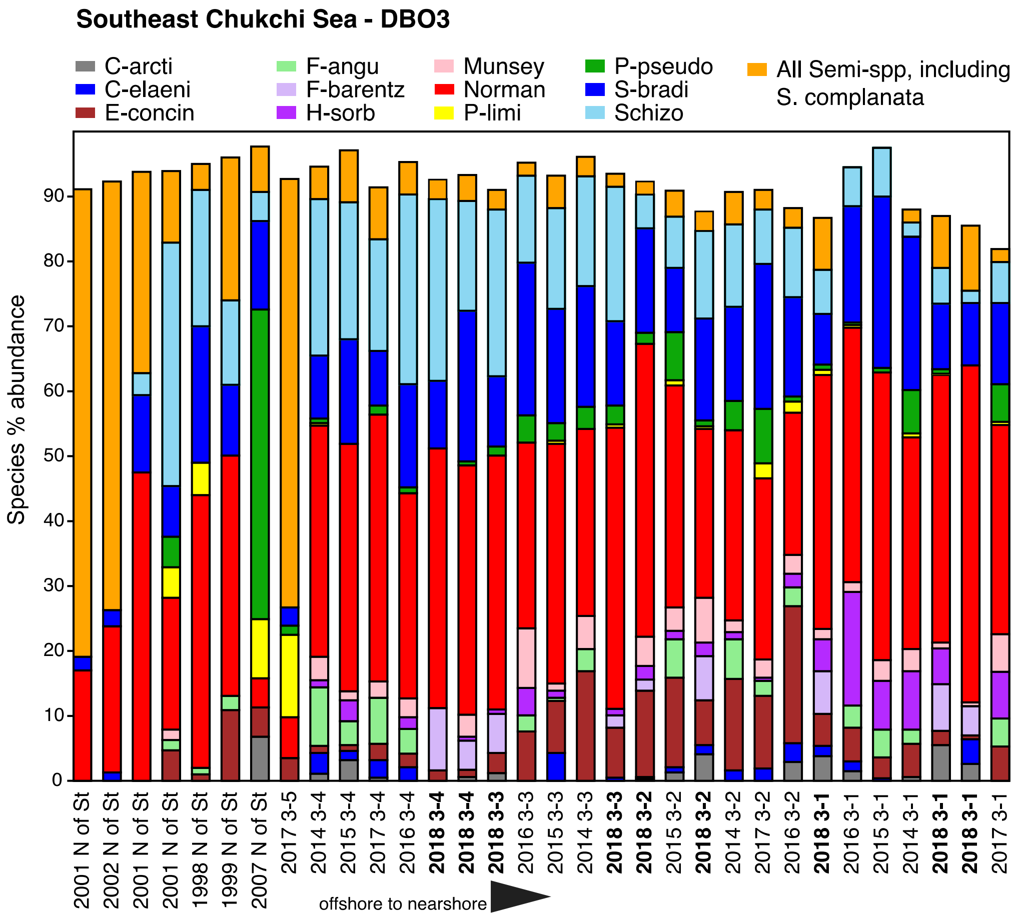

Fig 9

Primary species, Central Chukchi Sea – Ledyard Bay. Stack plot of the 12 most abundant species and one genus (*Semicytherura*), comprising >90% of the ostracode assemblage at the 2018 Ledyard Bay transect (sites on Fig. 2 map), southeast Chukchi Sea, (n=5 surface sediment samples, all with ≥50 specimens/sample, 558 total specimens).

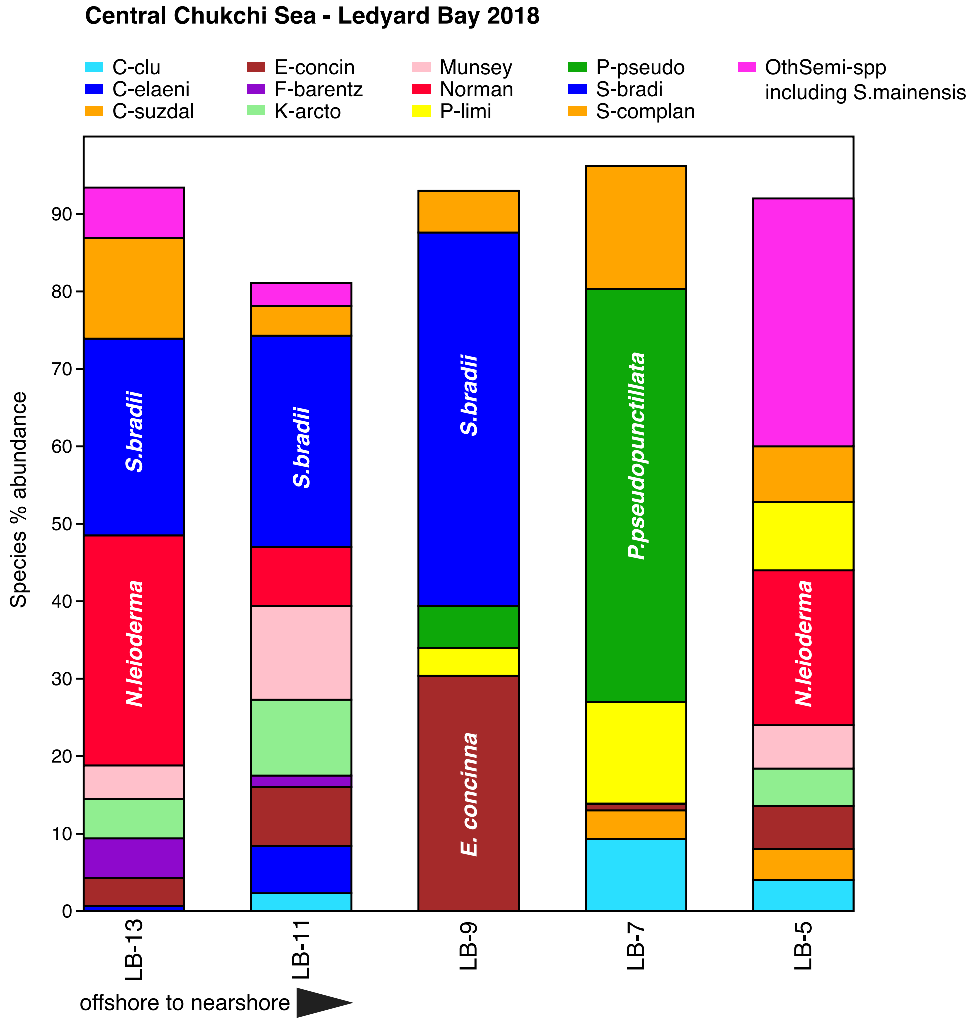

Fig 10

Primary species, Northeast Chukchi Sea - Icy Cape. Stack plot of the 9 most abundant species comprising ~90% of the ostracode assemblage at the 2018 Icy Cape (ICY) sampling transect (sites on Fig. 2 map), northeast Chukchi Sea, (n=10 samples with ≥75 specimens/sample [except ICY2 that contained 22 specimens], 1,554 total specimens). Species not included on plot that each represent >1-2% of transect assemblage total include *A. dunelmensis, Argilloecia* sp., and *E. concinna*.

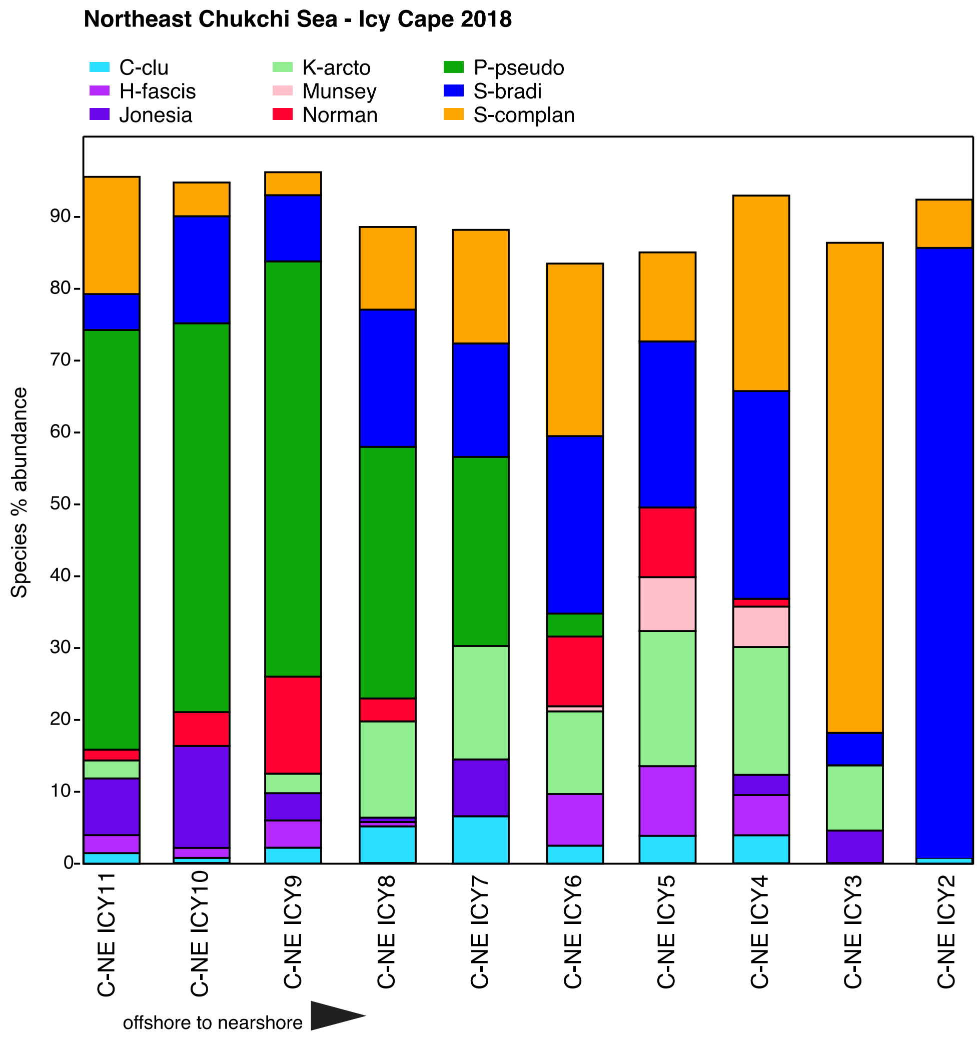

Fig 11

Primary species, Northeast Chukchi Sea – Hanna Shoal. Stack plot of the 11 most abundant species and one genus (*Argilloecia* spp.) comprising ~96% of the ostracode assemblage in the Hanna Shoal region offshore in the northeast Chukchi Sea (sites on Fig. 2 map) from years 2012 to 2013, (n=21 surface sediment samples with ≥30 specimens/sample, 2,647 total specimens in an average water depth of 51m).

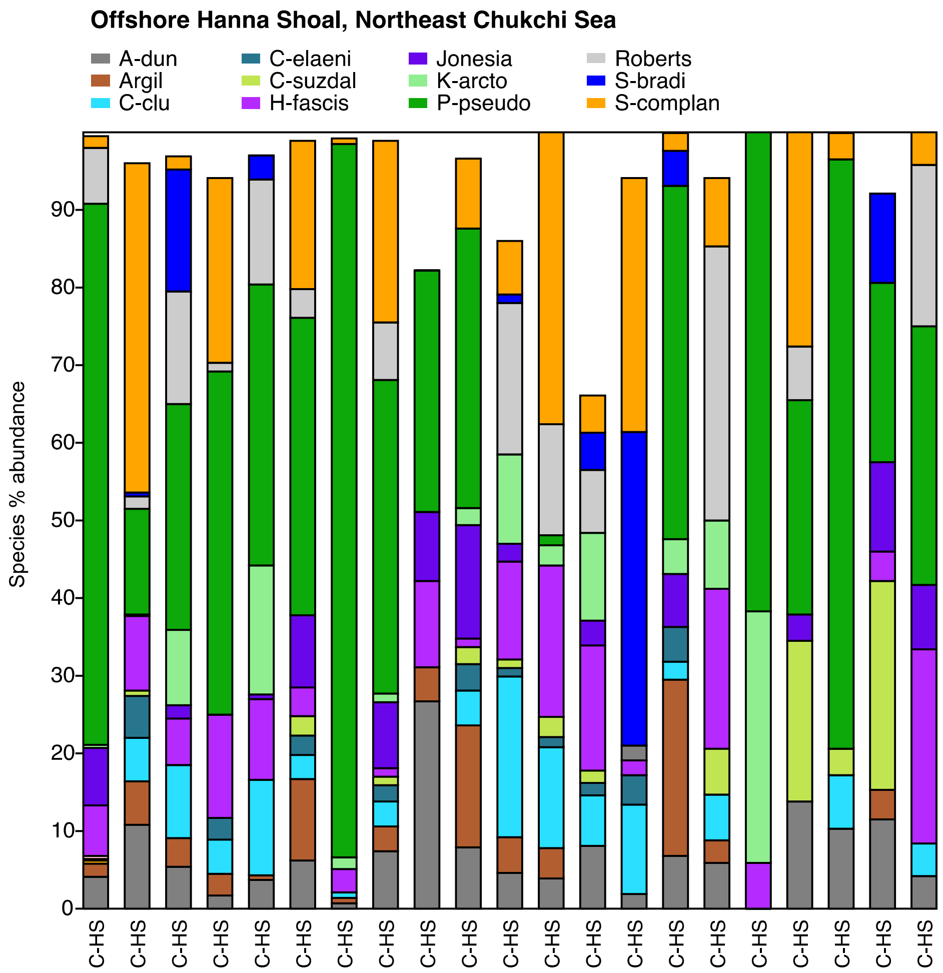

Fig 12

Primary species, DBO5 – Barrow Canyon. Stack plot of the 11 most abundant species and one genus (*Finmarchinella*), comprising ~80% of the ostracode assemblage, in DBO5-Barrow Canyon region (sites on Fig. 2 map) from years ranging from 2011 to 2018 (n=31 surface sediment samples with ≥30 specimens/sample, 2,951 total specimens). Other faunas at stations 5-9 and 5-10 that account for the species not shown on the stack plot include *A. dunelmensis, J. acuminata and H. fascis*, and *R. tuberculatus*.

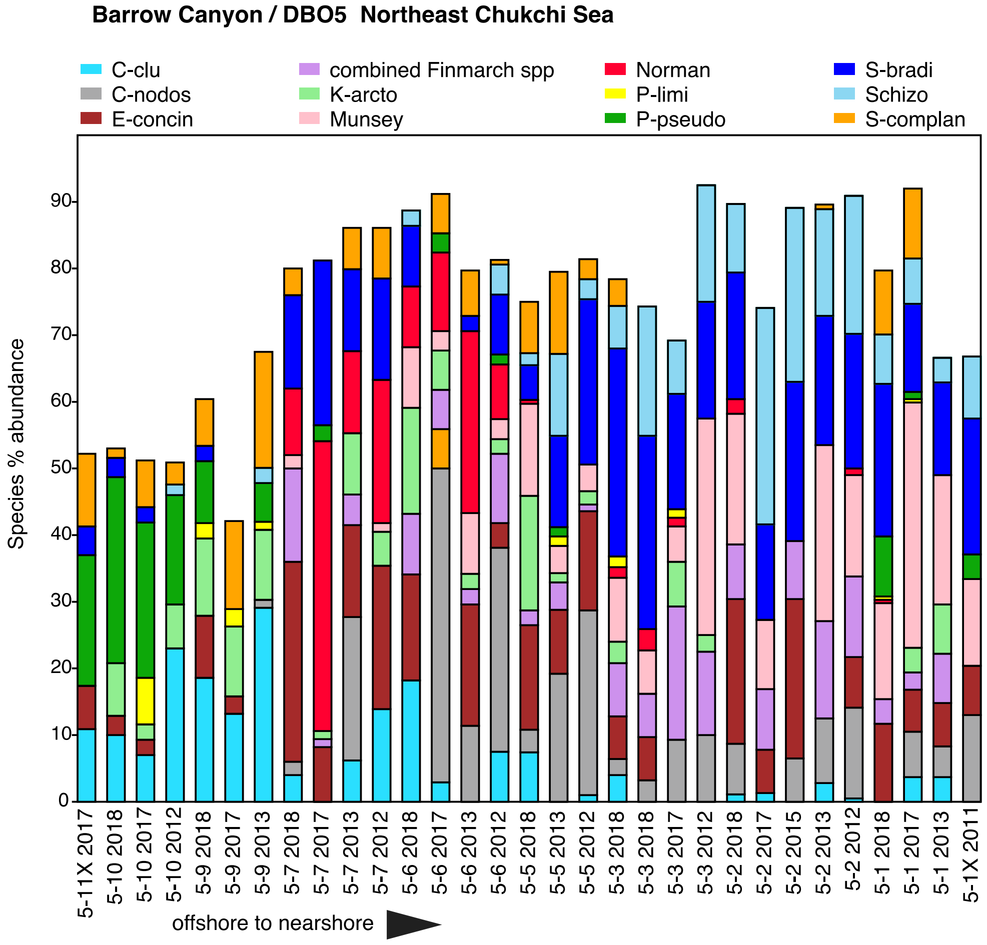

Fig 13

Primary species, Alaskan Beaufort Sea. Stack plot of the 12 most abundant species comprising ~85% of the ostracode assemblage in a narrow band of the Alaskan Beaufort Sea (sites on Fig. 4 map) within 100 km of shore at 20-90m water depth from years 1971 to 2018, (n=55 surface sediment samples with ≥30 specimens/sample, 6,617 total specimens). Samples collected in 2018 are highlighted in yellow. Other species not shown on the stack plot that account for 1-2% of the assemblage include *Rabilimis septentrionalis, Pteroloxa chaunensis, E. concinna*, and several *Cytheropteron* species. Important species in the Bering and Chukchi that exhibited either very low abundance (<1%) or are absent in the Beaufort Sea at 20-90m water depth are: *S. ikeyai., M. mananensis, Finmarchinella spp., K. arctoborealis, H. clathrata, H. emarginata, J. acuminata, S. affinis, S. mainensis*, and *S. undata*.

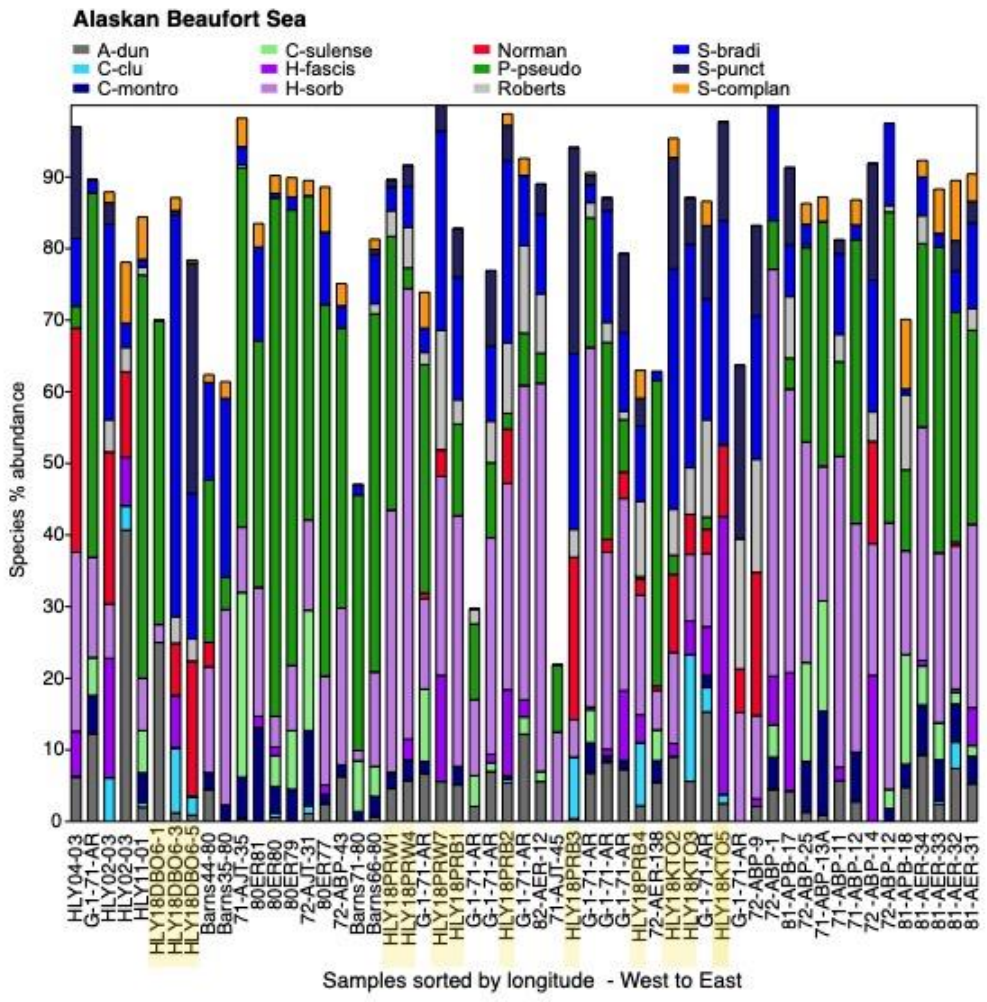

Fig 14

Principal correspondence analysis of ostracode assemblages by year groups. Synoptic PCAs using the biogeographic dataset (samples located on Fig 1 map) from the BCB grouped by the three regions and two collection periods, a.) 1970-2012 and b.) 2013-2018.

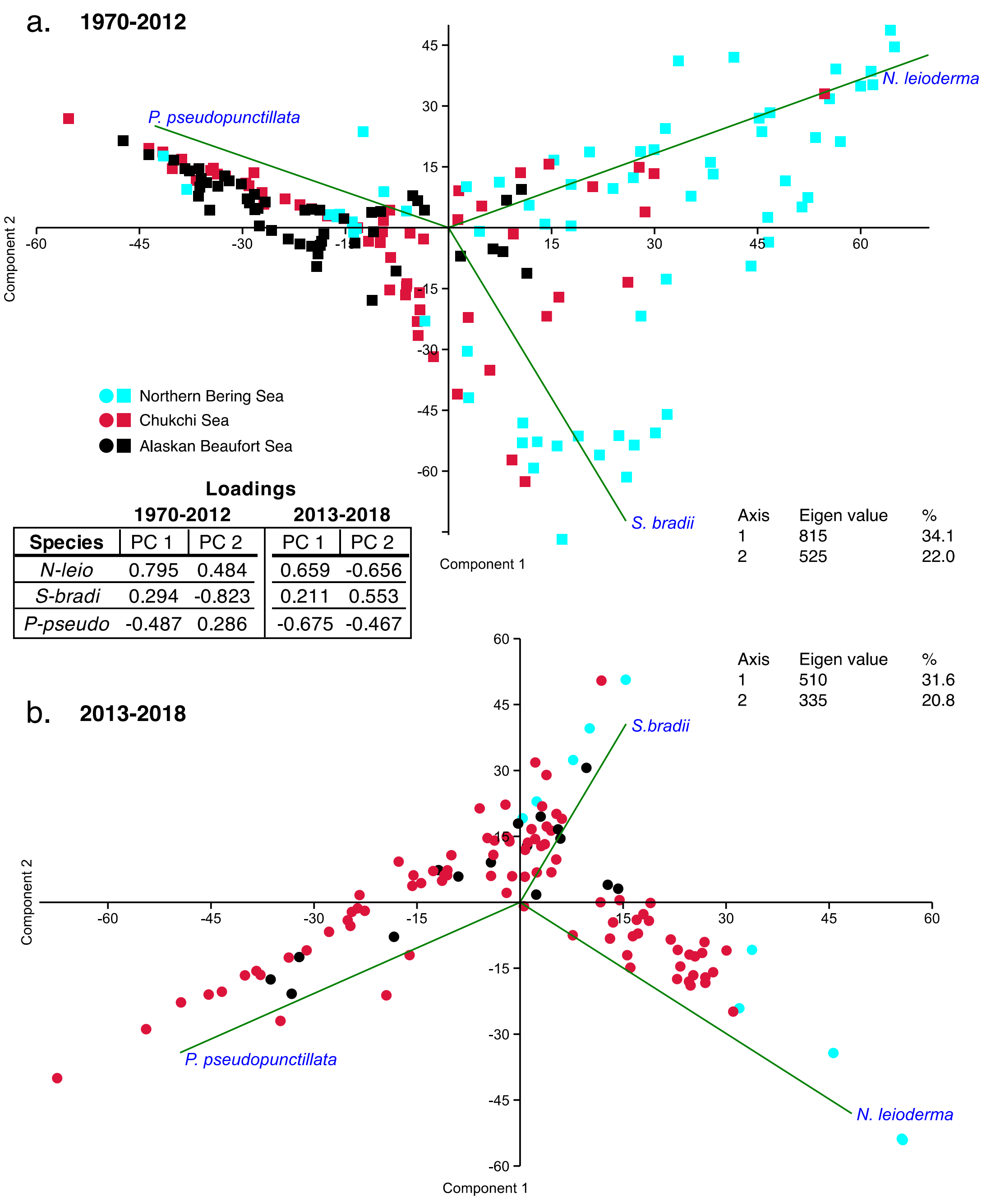

Loadings

Species	1970-2012 PC 1	1970-2012 PC 2	2013-2018 PC 1	2013-2018 PC 2
N-leio	0.795	0.484	0.659	-0.656
S-bradi	0.294	-0.823	0.211	0.553
P-pseudo	-0.487	0.286	-0.675	-0.467

Fig 15

Time series of key species in Chukchi Sea. a.) *N. leioderma*, b.) *P. pseudopunctillata*, c.) *S. bradii,* d.) *S. ikeyai*, e.) *M. mananensis*

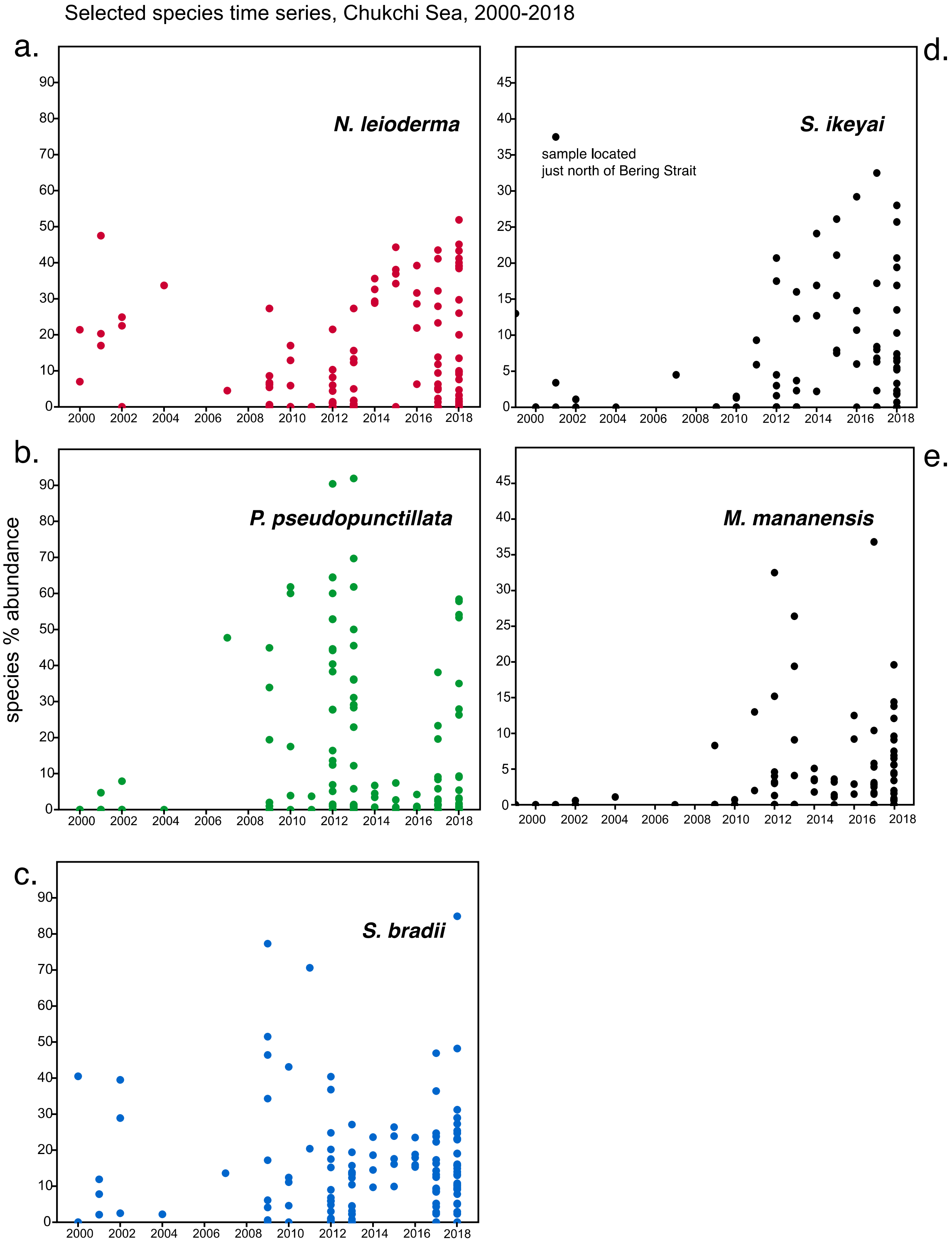

Table 1. **Ecoregion names and respective bounding coordinates of sample groups and transects investigated in this study.** Samples were grouped by ecoregions based on established Distributed Biological Observatory (DBO) station grids, which include four biological hotspot locations in the Bering and Chukchi Seas along a latitudinal gradient and sampled interannually: DBO1-south of St. Lawrence Island polynya (SLIP); DBO2-Chirikov Basin, north of St. Lawrence Island; DBO3-southern Chukchi Sea, southwest of Point Hope; and DBO5-Barrow Canyon in the northeast Chukchi Sea. Two other transects, sampled only in 2018, are also examined: Ledyard Bay (LB) and Icy Cape (ICY). Two other areas are defined in this study: northeast Chukchi (C-NE) that included sample locations in the northeast not specifically part of DBO4 or DBO5 transects; and Hanna Shoal (C-HS), an offshore region around Hanna Shoal. The Alaskan Beaufort Sea was treated as one ecoregion.
DBO station mean bottom water temperature, salinity, and sediment chlorophyll, with ± standard deviation, are provided for years 2000-2012, from March to July at DBO1, May to August at DBO2, July to Sept at DBO3, and May to Sept. at Northeast Chukchi. From Grebmeier et al., 2015a; Grebmeier and Cooper, 2014a and Okkonen, 2013. Ranges for sediment chlorophyll-a are provided for transects LB, ICY and DBO5 from the present study because values at a given location were very divergent from neighboring stations.

Ecoregions	DBO1 - South and west of St. Lawrence Island, Bering Sea	DBO2 - Chirikov Basin, Northern Bering Sea	DBO3 - Southern Chukchi Sea	Ledyard Bay (LB) transect, Chukchi Sea	Icy Cape (ICY) transect, Chukchi Sea	C-NE - Northeast Chukchi Sea	DBO5 - Barrow Canyon, Chukchi Sea	C-HS - Hanna Shoal, Chukchi Sea	Alaskan Beaufort Shelf
Latitude °N	63.77	65.111	68.60	70.30	71.83	71.00	71.808	73.30	70.60
°S	61.84	64.482	66.752	69.50	70.72	69.00	71.111	71.37	71.70
Longitude °E	-172.18	-167.86	-166.48	-165.40	-162.86	-159.40	-155.93	-158.30	-131.90
°W	-176.14	-170.49	-171.41	-168.50	-165.97	-173.80	-158.84	-165.60	-140.00
Depth range (m)	32-66	38-50	30-51	35-50	43-46	31-55	41-131	37-60*	20-90
Mean summer bottom temperature (°C)	-1.64 ±0.26	2.01 ±2.37	2.20 ±1.64	8.7 to 2.4	1.4 to -0.4	-0.70 ±1.54	0.1±2.3	-1.7	-0.9 ±0.7
Mean bottom salinity	32.47 ±0.32	32.2 ±0.61	32.36 ±0.56	31.7 ±0.53	32.1 ±0.1	32.59 ±0.84	32.6±0.9	33.0 ±0.3	31.4 ±1.0
Sediment chla (mg m-2)	13.31 ± 6.51	15.74 ± 8.15	19.16 ± 9.80	6.1 to 20.4	6.2 to 26.6	12.74 ± 7.73	5.2 to 38.6	8.7 ±4.1	not avail

Table 2. **List of 47 species and five genera found in the study region.** (≥1% of cumulative assemblage and/or occurred in 5 or more samples) Abbreviations of species are used in stack plots. According to the International Commission on Zoological Nomenclature, authors' names are in parentheses if the assignment listed was not the first description of the species.

A-dun	*Acanthocythereis dunelmensis* (Norman 1869) sensu lato
Argil	*Argilloecia* spp.
C-clu	*Cluthia cluthae* (Brady, Crosskey & Robertson 1874)
C-tesh	*Cytheretta teshekpukensis* Swain 1963
C-macch	*Cytheromorpha macchesneyi* (Brady and Crosskey 1871)
C-angul	*Cytheropteron angulatum* Brady and Robertson 1872
C-arcti	*Cytheropteron arcticum* Neale and Howe 1973
C-arcu	*Cytheropteron arcuatum* Brady, Crosskey & Robertson 1874
C-champ	*Cytheropteron champlainum* Cronin 1981
C-elaeni	*Cytheropteron elaeni* Cronin 1989
C-excav	*Cytheropteron excavoalatum* Whatley and Masson 1979
C-inflat	*Cytheropteron inflatum Brady*, Crosskey & Robertson 1874
C-montro	*Cytheropteron montrosiense* Brady, Crosskey & Robertson 1874
C-nodos	*Cytheropteron nodosoalatum* Neale and Howe 1973
C-perlaria	*Cytheropteron perlaria* Hao 1988
C-sulense	*Cytheropteron sulense* Lev 1972
C-suzdal	*Cytheropteron suzdalsky*i Lev 1972
C- tumefact	*Cytheropteron tumefactum* Lev 1972
Cyth-spp	*Cytheropteron* spp.
E-concin	*Elofsonella concinna* (Jones 1857)
E-neo	*Elofsonella neoconcinna* Bassiouni 1965
F-angu	*Finmarchinella angulata* (Sars 1866)
F-barentz	*Finmarchinella barentzovoensis* (Mandelstam 1957)
F-logani	*Finmarchinella logani* (Brady and Crosskey 1871)
H-emarg	*Hemicythere emarginata* (Sars 1866)
H-clathrata	*Hemicytherura clathrata* (Sars 1866)
H-fascis	*Heterocyprideis fascis* (Brady and Norman 1889)
H-sorb	*Heterocyprideis sorbyana* (Jones 1857)
Jonesia	*Jonesia acuminata* (Sars 1866) sensu lato
K-arcto	*Kotoracythere arctoborealis* Schornikov and Zenina 2006
K-hunti	*Krithe hunti* Yasuhara, Stepanova, Okahashi, Cronin and Brouwers 2014
Loxo	*Loxoconcha venepidermoidea* Swain 1963
Munsey	*Munseyella mananensis* Hazel and Valentine 1969
Norman	*Normanicythere leioderma* (Norman 1869)
P-limi	*Palmenella limicola* (Norman 1865)
P-pseudo	*Paracyprideis pseudopunctillata* Swain 1963
Paracytherois	*Paracytherois* spp.
P-chaun	*Pteroloxa chaunensis* Schornikov and Zenina 2006

R-mirab	*Rabilimis mirabilis* (Brady 1868)
R-septen	*Rabilimis septentrionalis* (Brady 1866)
Roberts	*Robertsonites tuberculatus* (Sars 1866)
Roundstonia	*Roundstonia globulifera* (Brady 1868)
S-bradi	*Sarsicytheridea bradii* (Norman 1865)
S-macro	*Sarsicytheridea macrolaminata* (Elofson 1939)
S-punct	*Sarsicytheridea punctillata* (Brady 1865)
Schizo	*Schizocythere ikeyai* Tsukagoshi and Briggs 1998
Sclero	*Sclerochilus* spp.
S-affinis	*Semicytherura affinis* (Sars 1866)
S-complan	*Semicytherura complanata* (Brady, Crosskey & Robertson 1874)
S-main	*Semicytherura mainensis* (Hazel and Valentine 1969)
Semi-spp	*Semicytherura* spp.
S-undata	*Semicytherura undata* (Sars 1866)

Table 3. DCA results based on the relative abundance of 49 ostracode species (including three genera, *Argilloecia, Cytheropteron* and *Semicytherura*) in 289 surface sediment samples from continental shelf regions of the Bering Chukchi and Beaufort Seas collected primarily in summer 1970-2018. Four assemblages related to average summer bottom water properties, and several species' preferences of bottom sediment texture, organic sediment food types, are established. Because this area is a broad continental shelf, there is overlap of taxa between Alaska Coastal Water and Bering Sea Water, which is bridged by *S. bradii* and secondary taxa that include *E. concinna*.

Group	Ecoregion	Distinguishing features	Typical bottom summer water mass*	Dominant species	Associated taxa in assemblage
**1**	Northern Bering Sea -- Western Chirikov Basin(DBO2), St. Lawrence Isl polyna	Subarctic-Arctic, polyna open water, high sediment chlorphyll-a, hot spot areas of high quality production, gravel-sandy sediments	Anadyr (-1.0 to 1.5°C, ≥32.5 salinity)	*Normanicythere leioderma*	*Sarsicytheridea bradii, Palmenella limicola, Semicytherura mainensis, Semicytherura affinis*
**2**	Nearshore Chukchi (DBO3-1 to 3-5 and DBO5-1 to 5-7)	Cold-temperate, subarctic, arctic; gravel to sandy sediments	Alaska Coastal Water (~ 3-9°C, ≥30 salinity)	*Normanicythere leioderma* and *Sarsicytheridea bradii*	*Schizocythere ikeyai, Munseyella mananensis, Semicytherura spp. (i.e. S. undata), Finmarchinella spp., Heterocyprideis sorbyana, Cytheropteron nodosalatum, Elofsonella concinna*
**3**	Eastern Chirikov Basin (DBO2) and central Bering Sea (middle shelf LB, ICY stations)	Arctic-subarctic; normal marine salinity	Bering Sea Water (0-3°C, 31.8-33.6 salinity)	*Sarsicytheridea bradii*	*Elofsonella concinna, Semicytherura complanata, Kotoracythere arctoborealis, Semicytherura affinis*
**3**	Offshore NE Chukchi and Hanna Shoal DBO5-9 and 5-10	Arctic, marine euryhaline, hot spot areas of high TOC detritus, fine-grained sediments	Bering Sea Water/ Winter Water (≤-1.0°C, ≤31.5 salinity avg. 33 salinity)	*Paracyprideis pseudopunctillata*	*Semicytherura complanata, Heterocyprideis fascis, Robertsonites tuberculatus, Acanthocythereis dunelmensis, Cluthia cluthae, Argilloecia spp., Kotoracythere arctoborealis, Jonesia acuminata*
**4**	Alaskan Beaufort Sea	Arctic, marine euryhaline, river influenced	Bering Sea Water/ Winter Water (≤-1.0°C, ≤31.5 salinity avg. 33 salinity)	*Paracyprideis pseudopunctillata* and *Heterocyprideis sorbyana*	*Sarsicytheridea bradii, Sarsicytheridea punctillata, Cytheropteron sulense, Heterocyprideis fascis, Acanthocythereis dunelmensis, Robertsonites tuberculatus*

S1 Table.

Six ecologically defined species in the northern Bering, Chukchi, and Beaufort Seas based on multivariate correlations with environmental factors. (*=from listed ecological references)

Species	ideal temperature	salinity	watermass	sediment substrate	food preference	representative climate zone	primary modern distribution*	mode of life	primary ecological reference(s)
Normanicythere leioderma	wide (-1.8 to 9°C)	normal marine (31-33)	AW, ACW, BSW	pebbly-sandy	newly settled production	subarctic-arctic / circum-Arctic	Sea of Japan, waters off eastern Japan, Bering Sea, polyna south of St. Lawrence Island (northern Bering), Chukchi Sea, Bay areas in North Atlantic (Gulf of St. Lawrence, Nova Scotia), Ellesmere Island, Iceland, northwestern Scotland, Gulf of Maine, Spitsbergen, Russian Harbor, Novaya Zemlya (Barents Sea)	epifaunal	Haz[illegible]; Ikeya and Cronin [illegible]993[illegible] Gemery et al., 2[illegible]is study
Paracyprideis pseudopunctillata	cold (≤1°C)	euryhaline	BSW, WW	fine silty muds	phytodetritis	arctic-subarctic / circum-Arctic	Beaufort Sea and nearshore waters off Greenland, Norwegian, Kara, Laptev, East Siberian, Norton Sound in Bering Sea, Chukchi Sea	infaunal	Stepanova, 2006
Sarsicytheridea bradii	eurythermic but more common in ≤4.5°C	normal marine	AW, ACW, BSW	wide	wide	arctic-subarctic / circum-Arctic	North Atlantic (north of Cape Cod and Georges Bank), Ungava Bay, Frobisher Bay, Hudson Bay, Gulf of Maine, straits of the Canadian Arctic Archipelago, Newfoundland, Labrador Sea, waters off Great Britain, Ireland, Norway, Greenland, Franz Josef Land, Spitsbergen, Novaya Zemlya, White, Baltic, North, Barents, Kara, Laptev, East Siberian, Chukchi nearshore waters off Aleutian Islands, Anadyr Bay, Bering, Norton Sound, nearshore waters off Alaska	infaunal	Stepanova, 20[illegible]eiwald and Mostafawi [illegible] Hazel 1970
Semicytherura complanta	cold (≤1°C)	normal marine, but high abundance in ≥33	BSW, WW	wide	unknown	arctic-subarctic / circum-Arctic	Areas where a polynya forms during wintertime; Greenland, White (southern inlet of the Barents Sea), Barents, Norwegian, Kara, Laptev, East Siberian, Chukchi, Bering, Beaufort Seas; Labrador seas, Ungava, Frobisher, and Hudson bays, straits of the Canadian Arctic Archipelago, Ireland	infaunal	Stepanova, 2006[illegible]ers et al., 2000; Cro[illegible]al. 1994
Schizocythere ikeyai	warm (0-20°C)	lower salinity areas <32	ACW	sandy	unknown	cold-temperate to subarctic	Waters off eastern Japan, Cook Inlet, Gulf of Alaska, Kodiak Shelf, Pribilof Islands, Norton Sound, Chukchi Sea	unknown	Tsukagosk[illegible]Briggs, 1998; Ozawa [illegible]; Cronin et al., 2020
Munseyella mananensis	warm (0-14°C)	lower salinity areas <32	ACW	sandy	unknown	cold-temperate to subarctic	Sea of Japan, Okhost Sea, Bering Sea, Gulf of Alaska; Ungava, Frobisher, Kneeland Bays off Canada and Baffin Bay off western Greenland, Halifax Inlet off Nova Scotia	unknown	Hazel and Valentine,1969; Schor[illegible]2001; Schornik[illegible] Zenina, 2006; Cronin et al., 2020

S1 Fig

a. Faunal abundance of selected taxa in relation to near-bottom temperature during summer sediment collection in the northern Bering and Chukchi Seas (n=211, 26,170 total specimens, 1990-2018, ≥30 specimens/sample).

b. Faunal abundance in relation to salinity.

c. Faunal abundance in relation to sediment type. Ostracode species abundance plotted against the percent sediment modal grain size of phi 0-4, where 0 represents gravel and rocks, 1 = coarse sand, 2 = medium sand, 3-4 = finer sand. Phi ≥5 (not shown) represents the very fine silty mud and clay sediment fraction typical of offshore or interior areas of the continental shelf.

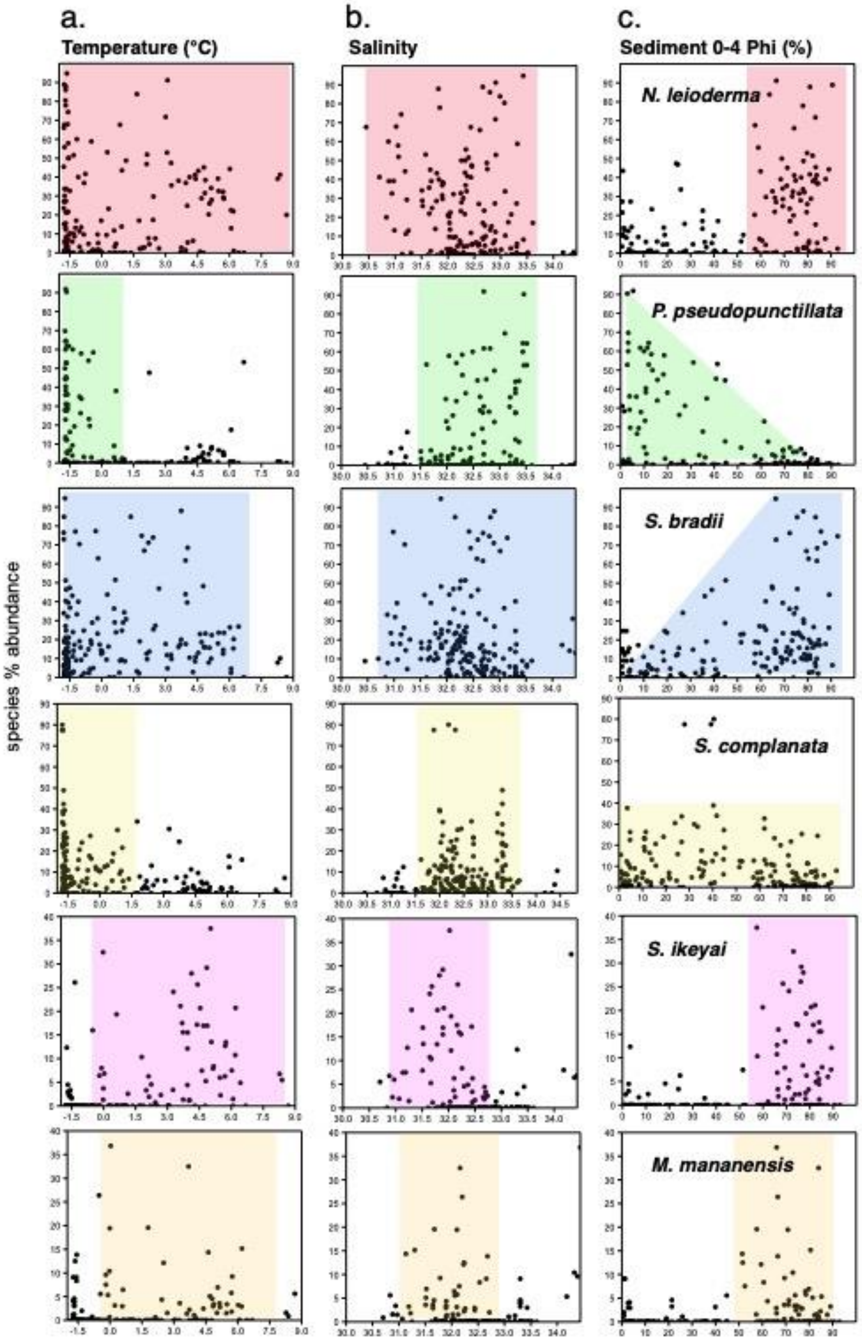

S2 Fig

Faunal abundance plotted against three different sources of sediment carbon, which may suggest preferred food sources: a. sediment chlorophyll-a (sedchla), b. total organic carbon (TOC) and c. carbon to nitrogen (C/N) ratios.

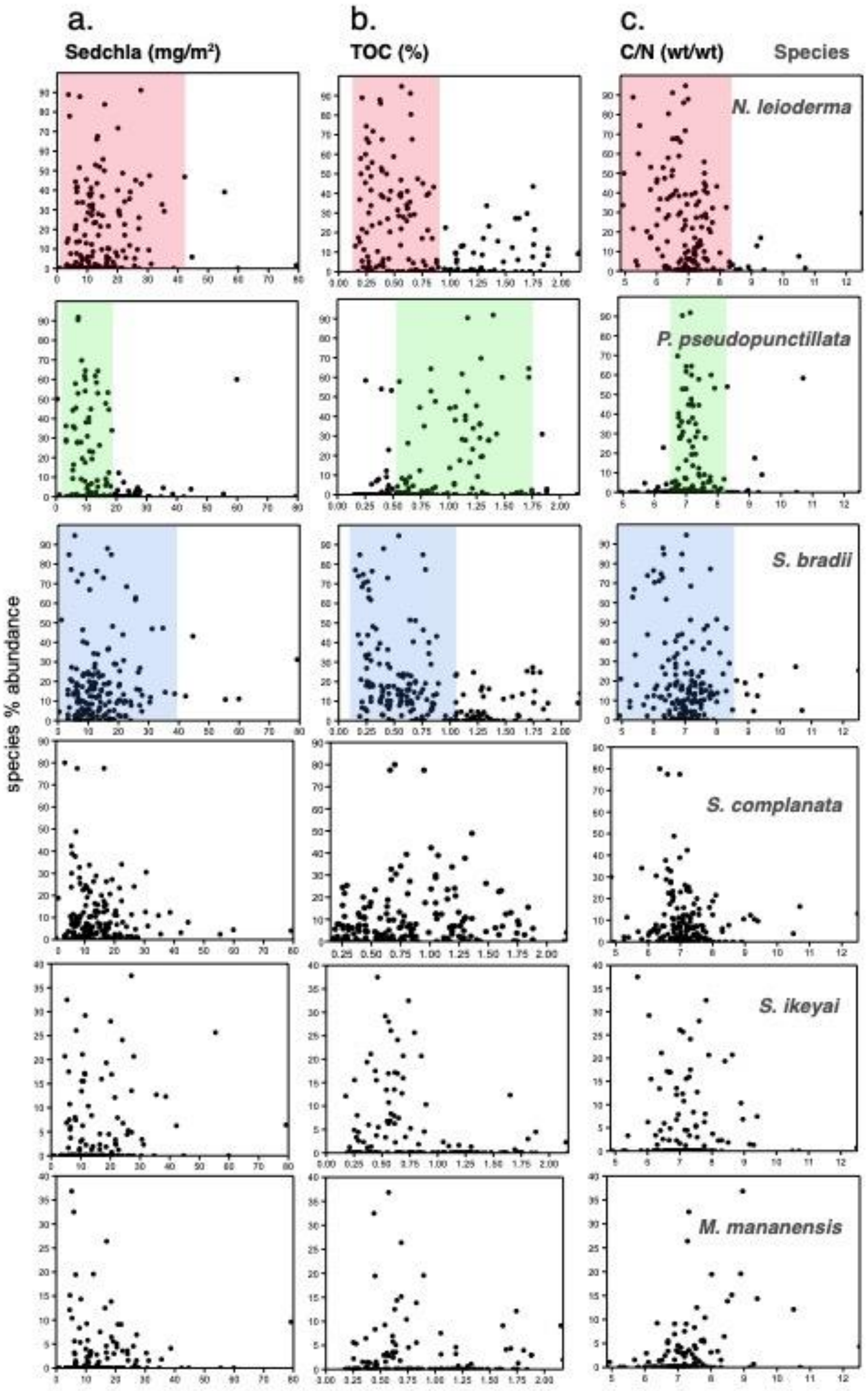

S3 Fig

Principal correspondence analysis (PCA) of Alaskan Beaufort Sea ostracode assemblages. Sample sites are designated by collection years (legend symbols) and major taxa (green lines with species names labeled in blue). The dominant species in the Alaskan Beaufort Sea are *H. sorbyana, P. pseudopunctillata, S. bradii* and, in samples collected in 2018, *N. leioderma*.

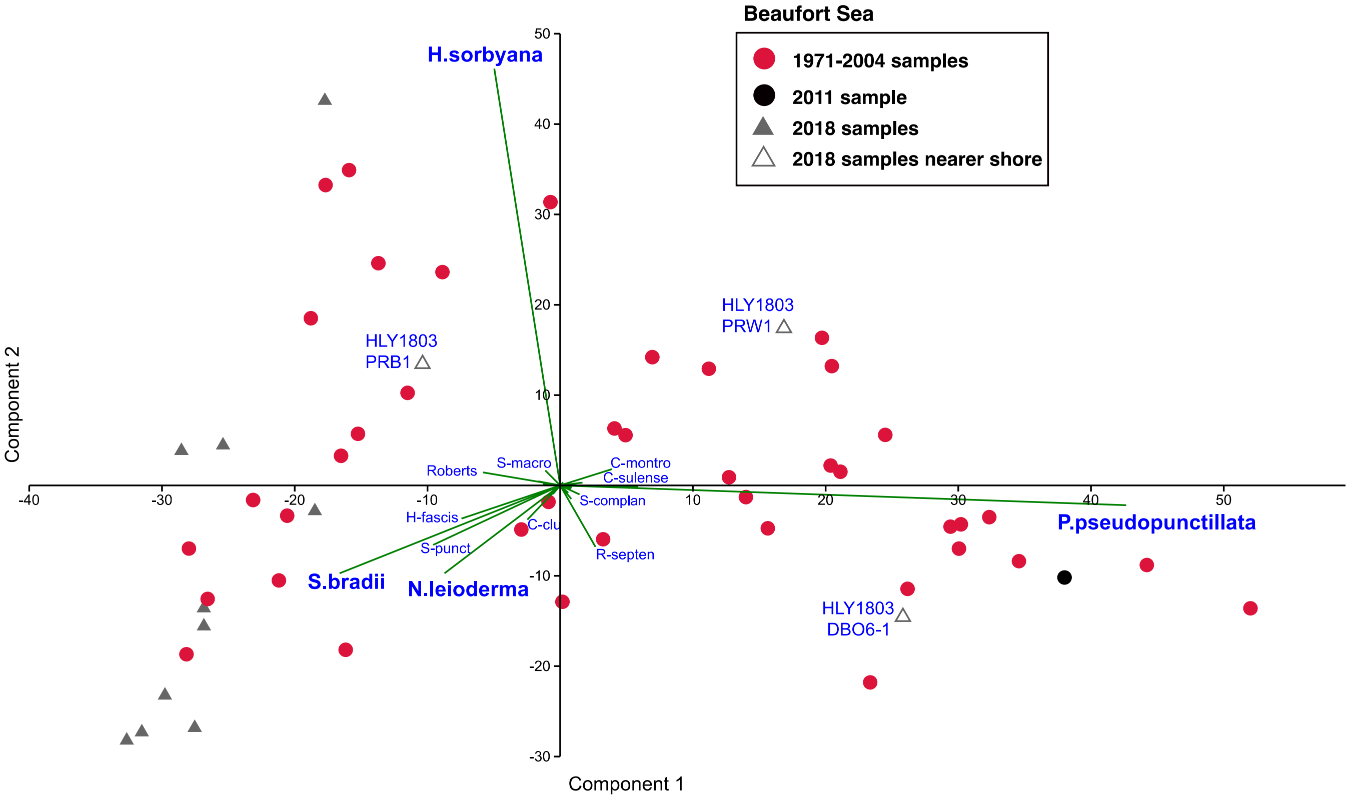

Supplement: Attachment — Submitted filename: PONE-D-21-04622_reviewer biblio.pdf [file pone.0251164.s006.pdf]
