# Supplementary material for: Biogeography and ecology of Ostracoda in the U.S. northern Bering, Chukchi, and Beaufort Seas
Source: PLoS One. 2021 May 13;16(5):e0251164. doi: 10.1371/journal.pone.0251164 (PMC8118254; doi:10.1371/journal.pone.0251164)
Supplement: Attachment — Submitted filename: Response to Reviewers - Fin.docx [file pone.0251164.s007.docx]

Reviewers' comments and Author’s response is indicated in RED text.

Reviewer's Responses to Questions

**Comments to the Author**

1. Is the manuscript technically sound, and do the data support the conclusions?

The manuscript must describe a technically sound piece of scientific research with data that supports the conclusions. Experiments must have been conducted rigorously, with appropriate controls, replication, and sample sizes. The conclusions must be drawn appropriately based on the data presented.

Reviewer #1: Yes

Reviewer #2: Yes

2. Has the statistical analysis been performed appropriately and rigorously?

Reviewer #1: Yes

Reviewer #2: Yes

3. Have the authors made all data underlying the findings in their manuscript fully available?

The [PLOS Data policy](https://gcc02.safelinks.protection.outlook.com/?url=http%3A%2F%2Fwww.plosone.org%2Fstatic%2Fpolicies.action%23sharing&data=04%7C01%7Clgemery%40usgs.gov%7C53a2e6f7bf154187e9cd08d8f9316cec%7C0693b5ba4b184d7b9341f32f400a5494%7C0%7C0%7C637533340051329688%7CUnknown%7CTWFpbGZsb3d8eyJWIjoiMC4wLjAwMDAiLCJQIjoiV2luMzIiLCJBTiI6Ik1haWwiLCJXVCI6Mn0%3D%7C0&sdata=1CpU94WtU%2B1Gb9wpb0K0F3%2F%2FUjXmrf6FJsMHubpw5s8%3D&reserved=0) requires authors to make all data underlying the findings described in their manuscript fully available without restriction, with rare exception (please refer to the Data Availability Statement in the manuscript PDF file). The data should be provided as part of the manuscript or its supporting information, or deposited to a public repository. For example, in addition to summary statistics, the data points behind means, medians and variance measures should be available. If there are restrictions on publicly sharing data—e.g. participant privacy or use of data from a third party—those must be specified.

Reviewer #1: Yes

Reviewer #2: Yes

4. Is the manuscript presented in an intelligible fashion and written in standard English?

PLOS ONE does not copyedit accepted manuscripts, so the language in submitted articles must be clear, correct, and unambiguous. Any typographical or grammatical errors should be corrected at revision, so please note any specific errors here.

Reviewer #1: Yes

Reviewer #2: Yes

5. Review Comments to the Author

Please use the space provided to explain your answers to the questions above. You may also include additional comments for the author, including concerns about dual publication, research ethics, or publication ethics. (Please upload your review as an attachment if it exceeds 20,000 characters)

**Reviewer #1:**

In my opinion, the paper is very interesting in approach and it furnishes relevant ostracod data from the Pacific-Arctic region, a key area of investigation also in the view of the climate change.
I’ve found especially interesting (i) the multivariate analyse of a big dataset composed of ca. 300 samples, collected during the last decades from continental shelves characterized by different physical-chemical parameters and (ii) the potentiality to use the obtained results to infer more robust paleoenvironmental reconstructions and to track potential changes in ecosystems under changing climate conditions.

Data interpretations and discussion are well justified and represent a logical product of a comprehensive and multi-faceted work.

I list below few comments, that I hope might be helpful to the Authors.

• I suggest to move the aims of the paper from lines 100-108 to the end of Introduction

Author’s response: We think this is a good suggestion, and have made this change

• Line 173: “≥5 phi = fine silts” maybe “fine silts and clay”?

Author’s response: Yes, we have added “and clay” here

• Was the ostracod matrix treated before the application of multivariate analyses?

Author’s response: No, there was no special treatment; The species counts were converted into species percent of the total assemblage and then used directly. The Bering-Chukchi-Beaufort dataset is a subset from the Arctic Ostracode Database-2020 (Cronin et al., 2021). As explained in the Methods section, we used only samples that contained ≥30 specimens, as that was the cutoff we deemed large enough to contain a representative sample of ostracodes living at a particular location.

• Concerning DCA (Fig. 6a), I wonder how the Authors traced the limits of the areas/groups and why a group of samples remained outside these groups, at the left edge of the biplot. I think that these points should be explained in text.

Author’s response: The bounding areas were drawn based on how the data naturally separated out and not any specific factoring program.

We’ve checked those samples on the far left that weren’t included in the ACW box, and thank the reviewer for bringing this to our attention. We have edited the figure bounding box and included those samples, which are affiliated with Alaska Coastal water.

• I suggest to integrate the section “Time-series analysis” into the discussion section “Temporal trends in BCB Sea indicator species”.

Author’s response: We consider the Time-series analysis to be part of the Results so the interpretation and implications are discussed in broader terms in the Discussion section, “Temporal trends.”

• Why any environmental gradient has been identified along the transect “Offshore Hanna Shoal” and “Alaskan Beaufort Sea”? I think that these points deserve a little bit of discussion.

Author’s response: We thank the reviewer for pointing this out and have added these points about the Hanna Shoal and Beaufort Sea to the respective sections: Both of these areas lack strong gradients. Hanna Shoal includes samples farther offshore around the shoal where sea ice usually persists longer, so that Shoal may have an influence based upon sea ice persistence. The transects across the Beaufort Sea represented a fairly uniform water mass because it represents 20-100m water depth, except closer to river inputs where *H. sorbyana* increased in abundance, as noted in the discussion. Also, these two areas were limited in sampling years since they have not been sampled consistently as part of the DBO sampling program. The Hanna Shoal samples were collected in 2012-2013 and the Beaufort Sea compared primarily in 2018 and 1971 with limited intermittent sampling in years in between.

• Figure 1: I suggest to insert a map for an easier localization of the studied area.

Author’s response: We have added an inset map to Fig. 1 for context of the study area.

• I suggest to add the ostracod matrix used for multivariate analyses as supplementary material.
Author’s response: Yes we have added this as an Excel file table to the supplementary material (Supplement Table 1).

**Reviewer #2:**

Dear Gemery and co-authors, your manuscript "Biogeography and ecology of Ostracoda in the U.S. northern Bering, Chukchi, and Beaufort Seas" is very well written and organised. I have annotated a pdf with some comments mainly about statistical analysis.

Author’s response:

As you will see in the tracked manuscript copy, we have made all the changes suggested by the Reviewer and also included comments in the pdf attachment. We have also added several references that were not included originally but cited in the text.

I suggest to add a plate with the indicator taxa and to revise the reference list.

Author’s response: We have added SEM images of these fauna to the Supplement Table 1 Figure.
